# Mechanistic adaptation of the metazoan RabGEFs Mon1-Ccz1 and Fuzzy-Inturned

**DOI:** 10.1126/sciadv.adx2893

**Published:** 2025-08-27

**Authors:** Stephan Wilmes, Jesse Tönjes, Maik Drechsler, Anita Ruf, Jan-Hannes Schäfer, Anna Lürick, Dovile Januliene, Steven Apelt, Daniele Di Iorio, Seraphine V. Wegner, Martin Loose, Arne Moeller, Achim Paululat, Daniel Kümmel

**Affiliations:** ^1^ Department of Chemistry and Pharmacy, Institute of Biochemistry, University of Münster, Münster, Germany.; ^2^ Department of Biology/Chemistry, Zoology section, Osnabrück University, Osnabrück, Germany.; ^3^ Department of Biology/Chemistry, Structural Biology section, Osnabrück University, Osnabrück, Germany.; ^4^ Center of Cellular Nanoanalytic Osnabrück (CellNanOs), Osnabrück University, Osnabrück, Germany.; ^5^ Institute of Physiological Chemistry and Pathobiochemistry, University of Münster, Münster, Germany.; ^6^ Institute of Science and Technology Austria (ISTA), Klosterneuburg, Austria.

## Abstract

Rab GTPases organize intracellular trafficking and provide identity to organelles. Their spatiotemporal activation by guanine nucleotide exchange factors (GEFs) is tightly controlled to ensure fidelity. Our structural and functional comparison of the tri-longin domain RabGEFs Mon1-Ccz1 and Fuzzy-Inturned reveals the molecular basis for their target specificity. Both complexes rely on a conserved sequence motif of their substrate GTPases for the catalytic mechanism, while secondary interactions allow discrimination between targets. We also find that dimeric Mon1-Ccz1 from fungi and the metazoan homologs with the additional third subunit RMC1/Bulli bind membranes through electrostatic interactions via distinct interfaces. Protein-lipid interaction studies and functional characterization in flies reveal an essential function of RMC1/Bulli as mediator of GEF complex membrane recruitment. In the case of Fuzzy-Inturned, reconstitution experiments demonstrate that the BAR (Bin-Amphiphysin-Rvs) domain protein CiBAR1 can support membrane recruitment of the GEF. Collectively, our study demonstrates the molecular basis for the adaptation of TLD-RabGEFs to different cellular functions.

## Teaser

The molecular comparison of related RabGEFs reveals adaptation mechanisms of a functional module for specific cellular tasks.

## INTRODUCTION

Organization of the eucaryotic endomembrane system critically depends on the precise spatiotemporal control of Rab guanosine triphosphatases (GTPases) (*1*). As markers of organelle identity and part of the conserved fusion machinery, Rabs are required for communication and exchange between different intracellular membrane compartments (*2*, *3*). They cycle between an active GTP (guanosine triphosphate)–bound form that associates with membranes via prenyl anchors and an inactive GDP (guanosine diphosphate)–bound form that is kept cytosolic through association with a GDI (guanosine dissociation inhibitor) chaperone. Depending on the nucleotide loading state, two switch regions of the Rab GTPase adopt different conformations that interact with different binding partners. The switch between both states is intrinsically slow and tightly controlled by regulatory proteins. GAPs (GTPase-activating proteins) stimulate GTP hydrolysis resulting in inactivation. Activation requires GEFs (guanine nucleotide exchange factors) that reduce the nucleotide affinity of the Rab GTPase and thus allow loading with GTP. Because the GTP-bound Rab can no longer bind to GDI, activation of Rabs simultaneously leads to its membrane recruitment. The localization of RabGEFs therefore also determines the subcellular distribution of their cognate Rab GTPases and represents a key event in achieving specificity in intracellular trafficking.

**Fig. 1.**
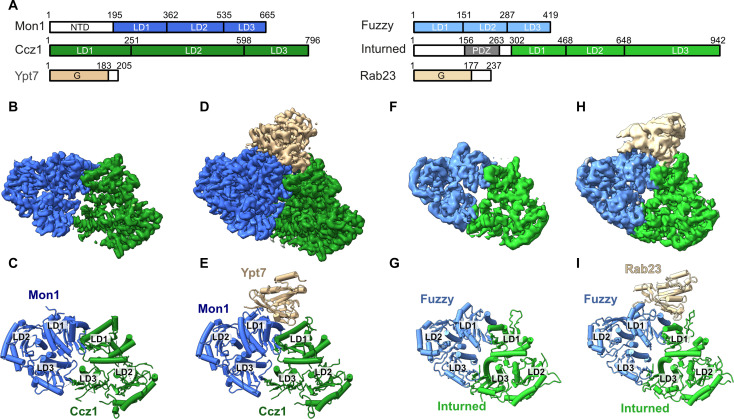
Structural analysis of TLD GEF-Rab complexes. (**A**) Domain architecture of *Ct*Mon1, *Ct*Ccz1, *Ct*Ypt7, *Hs*Fuzzy, *Hs*Inturned, and *Hs*Rab23. LD, longin domain; G, GTPase domain; PDZ, PSD95/Dlg1/ZO-1 domain. (**B**) Cryo-EM map and (**C**) cartoon representation of the dimeric *Ct*Mon1-Ccz1^ΔL^ complex. (**D**) Cryo-EM map and (**E**) cartoon representation of the trimeric *C*tMon1-Ccz1^ΔL^-Ypt7^N125I^ complex. (**F**) Cryo-EM map and (**G**) cartoon representation of the dimeric *Hs*Fuzzy-Inturned complex. (**H**) Cryo-EM map and (**I**) cartoon representation of the trimeric *Hs*Fuzzy-Inturned-Rab23^N121I^ complex.

The tri-longin domain (TLD) RabGEF complexes represent a family of RabGEFs with a unique conserved structural core (*4*, *5*). In each complex, two subunits that contain three longin domains (LD1 to LD3) each form a heterodimer, which is required for catalytic activity toward the cognate Rab GTPases. Different TLD-GEFs vary in their composition as they can contain additional domains or bind auxiliary subunits (Fig. 1A).

The Mon1-Ccz1 complex is the best-studied TLD GEF and conserved throughout all eukaryotes (*6*–*11*). Mon1-Ccz1 is a dimer in yeast and activates the Rab7 homolog Ypt7. This process is required in endosomal maturation and autophagy, where generation of Ypt7-positive compartments is destined for fusion with the vacuole, the lysosome-equivalent organelle of yeast (*12*). Structural and functional studies have provided a framework for a mechanistic understanding of Mon1-Ccz1 (*13*–*16*). The first longin domains of Mon1 and Ccz1 form the active site and bind Ypt7 to remodel switch 1 of the GTPase (*15*). As a consequence, a conserved lysine is inserted into the nucleotide-binding pocket and expels the essential magnesium cofactor, leading to lowered nucleotide affinity. The localization of Mon1-Ccz1 to endosomes requires binding to active Rab5-like GTPases and binding to phosphatidylinositol phosphate (PIP). Autophagosomal recruitment, however, relies on the interaction of Mon1-Ccz1 with Atg8 and with lipid packing defects. Thus, Mon1-Ccz1 is differentially targeted by distinct molecular cues (*17*). In metazoans, Mon1-Ccz1 form a trimer with an additional subunit Bulli/RMC1 and control Rab7 activation, fulfilling a conserved function in organizing lysosomal fusion (*7*, *8*, *10*). Recent cryo–electron microscopy (cryo-EM) structures of the human Mon1-Ccz1-RMC1 complex with the substrate Rab7 confirmed that the catalytic mechanism is conserved in fungi and metazoans and does not involve a contribution from Bulli/RMC1 (*18*, *19*). Thus, while Bulli/RMC1 has been shown to be functionally important in vivo, its molecular role remains elusive.

The BLOC-3 (biogenesis of lysosome-related organelles complex-3) complex, composed of the TLD subunits Hps1 and Hps4, is unique to metazoans and activates Rab32 and Rab38 for the maturation of lysosome-related organelles (*20*). Mutations in the *HPS1* and *HPS4* genes cause the genetic disease Hermansky-Pudlak syndrome, but the molecular basis underlying BLOC-3 function is poorly understood (*21*). The cryo-EM structures of BLOC-3 and a complex with its recruiter Rab9A provide an important molecular framework for further insight (*22*).

Last, the TLD proteins Fuzzy and Inturned act as GEF for Rab23 (*5*). In a trimeric complex with the protein Fritz, Fuzzy-Inturned constitute the PPE (planar cell polarity effector) complex in *Drosophila melanogaster* (*Dm*) and are involved in establishing planar cell polarity (*23*). In mammalian cells, Inturned, Fuzzy, the Fritz homolog Wdpcp and the atypical GTPase Rsg1 form the CPLANE (ciliogenesis and planar cell polarity effector) complex that is required for cilia formation (*24*). The molecular understanding of Fuzzy and Inturned function remains incomplete. A reported cryo-EM structure of CPLANE established structural similarity between Fuzzy-Inturned and Mon1-Ccz1 (*25*). Furthermore, binding to PIP lipids has been suggested for several CPLANE subunits, but it is not clear how the complex is regulated and localized in cells.

We here report the cryo-EM structures of the Mon1-Ccz1 and Inturned-Fuzzy core complexes bound to their cognate GTPases Ypt7 and Rab23 in the nucleotide exchange transition states, revealing a conserved catalytic mechanism with distinct modes of substrate recognition. Furthermore, we quantitatively show that fungal Mon1-Ccz1 robustly interacts with lipids, whereas metazoan Mon1-Ccz1 and Inturned-Fuzzy complexes do not. Instead, metazoan TLD-GEFs depend on binding to accessory subunits and recruiter proteins. For CPLANE, we demonstrate that the BAR (Bin-Amphiphysin-Rvs) domain adaptor protein CiBAR1/FAM92A1 (*26*–*28*) can fulfill this role. These findings advance our understanding of Rab regulation by TLD RabGEFs in metazoans, which are relevant fundamental physiological processes like ciliogenesis and related pathological scenarios.

## RESULTS

### Structural analysis of substrate-bound Mon1-Ccz1 and Fuzzy-Inturned

TLD RabGEFs have been proposed to use a conserved catalytic mechanism but reportedly maintain a high specificity for their respective substrate GTPases (*5*, *15*). To provide the molecular basis of these observations, we determined the cryo-EM structures of fungal Mon1-Ccz1 from *Chaetomium thermophilum* (*Ct*) bound to nucleotide-free Ypt7 and of human Fuzzy-Inturned in complex with nucleotide-free Rab23.

In our previous work, we resolved a crystal structure of a catalytic *Ct*Mon1^LD1^-Ccz1^LD1^-Ypt7^N125I^ subcomplex (*15*) and the cryo-EM structure of a truncated *Ct*Mon1^ΔN^-Ccz1^ΔL^ complex lacking the N-terminal domain (NTD) of Mon1 (residues 1 to 140) and the lipid-binding loop of Ccz-1 (L, residues 333 to 444) (*13*). However, the N-terminal region of Mon1 was shown to play a role in regulating the complex activity (*29*). Furthermore, it has been shown that some Rab GEF complexes like TRAPP (*30*) or Ric1-Rgp1 (*31*) have high affinity binding sites for the hypervariable domain (HVD) of their substrate GTPases. Recognition of secondary motifs in addition to G domain binding has emerged as an important factor for Rab specificity (*30*–*33*). To investigate potential interactions of Mon1^NTD^ and Ypt7^HVD^ with the TLD GEF core complex, we use full-length Mon1 and Ypt7 in the current study. To support the formation of a stable transition state of the complex, the nucleotide-free variant *Ct*Ypt7^N125I^ was coexpressed with *Ct*Mon1 and *Ct*Ccz1^ΔL^ in *Escherichia coli*. Analysis of purified complexes by cryo-EM revealed a mixture of dimeric *Ct*Mon1-Ccz1 and trimeric *Ct*Mon1-Ccz1-Ypt7 particles. We could determine the structure of both complexes at 3-Å resolution (Fig. 1, B to E, and figs. S1 to S3, A and B). The structure of the dimeric complex with full-length Mon1 closely resembles the structure of truncated Mon1^ΔN^-Ccz1^ΔL^ [root mean square deviation (RMSD) of 1.17 Å over 837 residues; fig. S3C], but the higher resolution of the current structure allowed us to improve the sequence assignment of the final β strand of Ccz1. In the trimeric complex, the structures of Mon1 and Ccz1 remain virtually unchanged (RMSD, 0.67 Å; Fig. 1), and we assigned the additional density to the G domain of Ypt7. We did not observe structural changes in binding of Ypt7 to intact Mon1-Ccz1 in comparison to the Mon1^LD1^-Ccz1^LD1^ subcomplex (RMSD of 1.03 Å over 436 residues). The experimental map does not reveal unmodeled density that could be interpreted as the Mon1^NTD^ or Ypt7^HVD^. Consequently, our structures do not provide support for models that suggest an autoinhibitory interaction of the NTD of Mon1 with the longin domains or recognition of the Ypt7^HVD^ by Mon1-Ccz1 at the chosen conditions. However, these interactions may require the presence of membranes or recruiter proteins.

To resolve the *Hs*Fuzzy-Inturned-Rab23 complex, we again used a nucleotide-free mutant of the GTPase (Rab23^N121I^) and performed coexpression in insect cells. From the analysis of the cryo-EM dataset, we could determine the structure of the dimeric Fuzzy-Inturned complex in addition to the trimeric *Hs*Fuzzy-Inturned-Rab23^N121I^ complex at 3.6- and 3.4-Å resolution, respectively (Fig. 1, F to I, and figs. S4 to S6, A and B). The conformations of Fuzzy and Inturned do not change upon Rab23 binding (RMSD, 0.82 Å; Fig. 1) and are virtually identical compared to the full CPLANE complex that also includes Rsg1 and Wdpcp (RMSD of 1.25 Å over 748 residues; fig. S6C) (*25*). All longin domains of Fuzzy and Inturned are well resolved, but as in CPLANE, an N-terminal PDZ domain of Inturned is not visible in the map.

**Fig. 2.**
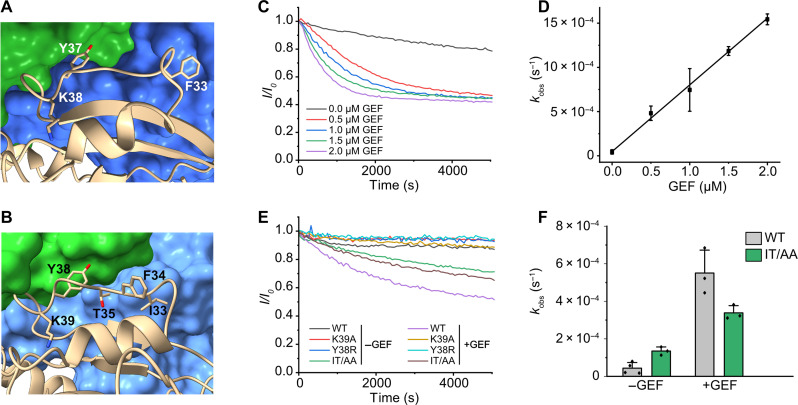
Comparison of the catalytic GEF mechanism. (**A**) Close-up of the Ypt7 switch 1 region (tan) interacting with Mon1 (blue) and Ccz1 (green). (**B**) Close-up of the Rab23 switch 1 region (wheat) interacting with Fuzzy (blue) and Inturned (green). (**C**) Guanine nucleotide exchange assay of Rab23 with different concentrations of Fuzzy^LD1^-Inturned^LD1^ catalytic core complex. (**D**) Determination of catalytic efficiency from *n* = 3 independent experiments as in (C). Data are represented as means ± SD. (**E**) Guanine nucleotide exchange assay of Rab23 wild-type (WT) and different variants (IT/AA: I33A and T35A) with and without Fuzzy^LD1^-Inturned^LD1^. (**F**) Intrinsic and guanine nucleotide exchange factor (GEF)–stimulated nucleotide exchange rates of Rab23 WT and the IT/AA variant from *n* = 3 repeats. Data are represented as means ± SD.

The GTPases interact in a very similar orientation with same interfaces of the GEF complexes, which are provided by the first longin domains of the TLD subunits (Figs. 1, E and I, and 2, A and B). Fuzzy and Mon1 fulfill homologous functions and contribute most of the interface with Rab23 (885 Å^2^) and Ypt7 (1270 Å^2^), respectively. However, Inturned and Ccz1 also both make critical contacts with the switch 1 regions (428 and 469 Å^2^, respectively). Overall, the structures of Mon1-Ccz1-Ypt7 and Inturned-Fuzzy-Rab23 complexes show a highly similar architecture (RMSD of 2.59 Å over 747 aligned residues).

### Conserved and variable features of the TLD RabGEF mechanism

Remodeling of the Ypt7 switch 1 region by Mon1-Ccz1 causes an opening of the nucleotide binding pocket, removal of a guanine base interacting phenylalanine (F33), and the insertion of a catalytic lysine residue (K38) in the binding site that expels the magnesium cofactor (Fig. 2A). F33 and a tyrosine (Y37) adjacent to the catalytic lysine bind to hydrophobic pockets on Mon1-Ccz1, thus stabilizing the switch 1 conformation. Collectively, this structural arrangement was shown to lower the nucleotide affinity of Ypt7 and thus promote nucleotide exchange (*15*).

A basic residue at the position of the catalytic lysine is conserved in the Inturned-Fuzzy substrate Rab23 (K39) and the BLOC-3 substrates Rab32 and Rab38 (R55 and R38, respectively), as well as the adjacent tyrosine (fig. S7A). This suggested that the catalytic mechanism of TLD RabGEFs is likely conserved. When bound to its GEF, K39 and Y38 of Rab23 adopt the same conformation as the corresponding residues of Ypt7 (Fig. 2B). We used an in vitro GEF assay with a Fuzzy^LD1^-Inturned^LD1^ core complex, which showed robust nucleotide exchange activity on Rab23 wild type (WT) (catalytic efficiency *k*_cat_*/K*_M_ of 7 × 10^2^ M^−1^ s^−1^; Fig. 2, C and D). However, upon mutation of either of the conserved residues K39 to alanine or Y38 to arginine, Rab23 cannot be stimulated by Fuzzy-Inturned (Fig. 2E). The corresponding residues in the BLOC-3 substrates Rab32 (Y54 and R55) and Rab38 (Y38 and R39) were also shown to be required for GEF activity (*22*). Thus, the common YK motif in TLD RabGEF substrates is part of a conserved mechanism. The switch 1 region of Rab23 is well resolved in the cryo-EM map (fig. S6B), and variations in the GEF interaction compared to Ypt7 can be observed that likely encode substrate specificity. The guanine base stabilizing aromatic residue F34 of Rab23 is removed from the binding pocket, but in contrast to F33 of Ypt7, it does not bind to a hydrophobic pocket on the GEF surface. Instead, residues I33 and T35 interact with Inturned and thus fix the switch 1 conformation (Fig. 2B). A double mutant Rab23^I33A/T35A^ showed increased intrinsic nucleotide exchange but reduced stimulation of nucleotide exchange by Fuzzy-Inturned (Fig. 2, E and F). Because structures of Rab23 in the GTP- and GDP-bound states (*34*) showed that I33 and T35 do not interact with the nucleotide directly (fig. S7, B and C), the increased intrinsic exchange rate is likely caused by a destabilization of switch 1. However, the ~5-fold diminished ability of Fuzzy-Inturned to promote nucleotide exchange of Rab23^I33A/T35A^ confirms the importance of these amino acids for recognition by the GEF and for the catalytic function. Thus, TLD RabGEFs rely on both conservation and variability in the switch 1 regions of their substrates to realize catalytic efficiency and specificity.

### The metazoan Mon1-Ccz1-Bulli complex binds negatively charged membranes

**Fig. 3.**
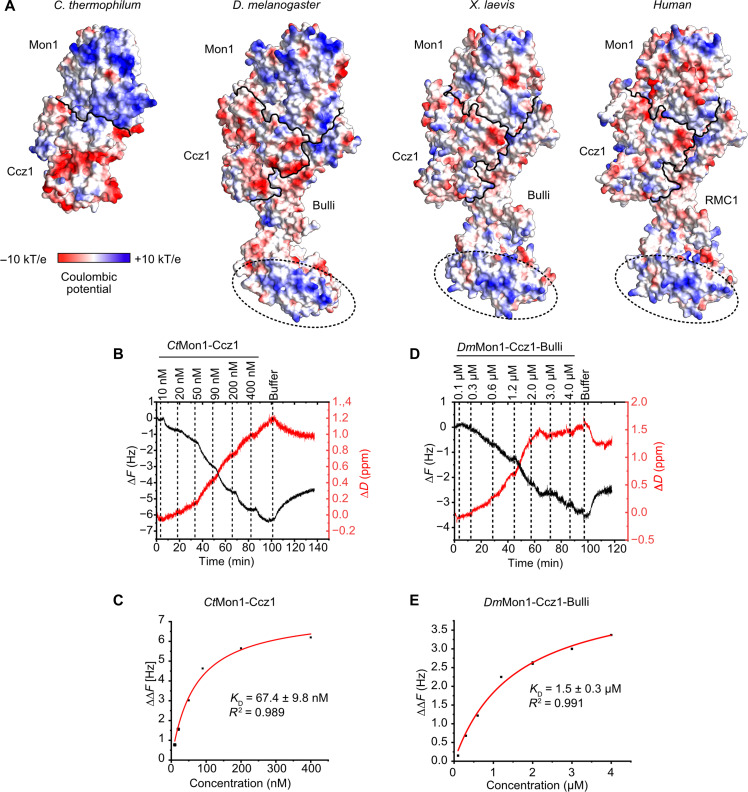
Membrane binding of fungal and metazoan Mon1-Ccz1 complexes. (**A**) Surface representation of the lipid interaction side *Ct*Mon1-Ccz1 and of *Dm*Mon1-Ccz1-Bulli, *Hs*Mon1-Ccz1-RMC1, and *Xl*Mon1-Ccz1-Bulli in the corresponding orientation. Coloring is by electrostatic potential. Subunit boundaries are indicated by black lines, and a conserved basic patch on Bulli/RMC1 is highlighted by circles. (**B**) QCM-D measurements of a *Ct*Mon1-Ccz1 titration on a supported lipid bilayer (74 mol% DO-PC, 18 mol% DO-PE, 2 mol% DO-PI3P, 1 mol% DO-PI(3,5)P_2_, and 5 mol% DO-PS). (**C**) QCM-D frequency shifts of *Ct*Mon1-Ccz1 plotted against the concentration of protein were fitted with a one-step binding model for *K*_D_ determination. (**D**) QCM-D measurements of a *Dm*Mon1-Ccz1-Bulli titration on a supported lipid bilayer as in (B). (**E**) QCM-D frequency shifts of *Dm*Mon1-Ccz1-Bulli plotted against the concentration of protein were fitted with a one-step binding model for *K*_D_ determination. ppm, parts per million; *R*^2^, coefficient of determination.

In addition to substrate recognition, spatiotemporal control of RabGEF localization has been recognized as a key mechanism for their specificity and regulation (*1*, *32*). The analysis of the electrostatic surface potential of Mon1-Ccz1 revealed a large basic patch on the Mon1^LD2^ located opposite of the Ypt7 binding site that—together with an amphipathic helix in a disordered region of Ccz1^LD2^ (*17*)—mediates binding of Mon1-Ccz1 to model membranes (Fig. 3A). For a quantitative determination of membrane affinity, we turned to QCM-D (quartz crystal microbalance with dissipation monitoring) measurements. Supported lipid bilayers (SLBs) were formed with a DO/PIP/PS lipid mix that displays packing defects and negative charges (fig. S8A). Here, the titration of *Ct*Mon1-Ccz1 on the SLBs yielded an apparent dissociation constant *K*_D_ of 67 nM (Fig. 3, B and C).

**Fig. 4.**
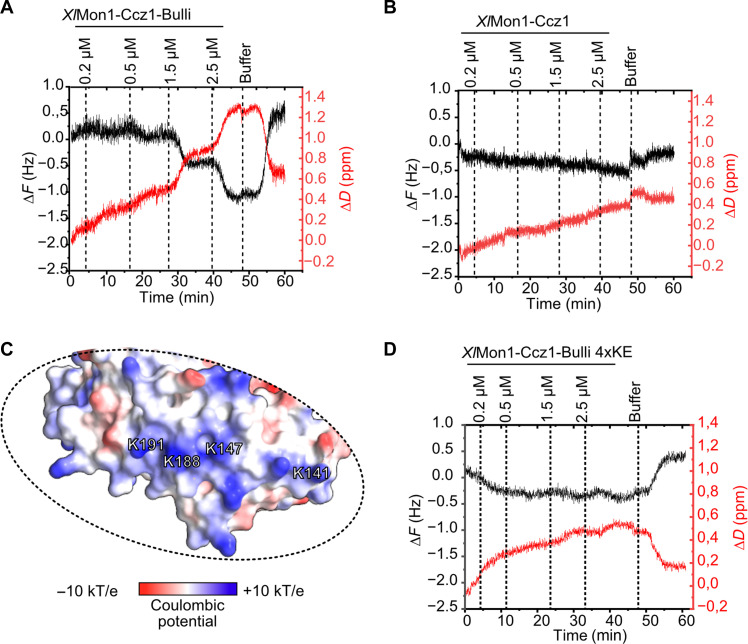
Membrane binding is mediated by Bulli in vitro. (**A**) QCM-D measurements of a *Xl*Mon1-Ccz1-Bulli titration on a supported lipid bilayer as in Fig. 3. (**B**) QCM-D measurements of a *Xl*Mon1-Ccz1 titration on a supported lipid bilayer as in (B). (**C**) Close-up of the basic patch of *Xl*Bulli corresponding to the region marked in Fig 3A. Four lysine residues that map to the patch are labeled. (**D**) QCM-D measurements of a *Xl*Mon1-Ccz1-Bulli 4xKE titration on a supported lipid bilayer as in Fig. 3.

The importance of lipid recognition via a basic patch and amphipathic helix in conjunction with recruiter proteins for the function of Mon1-Ccz1 in fungi has been established in vitro and in cellular studies with yeast (*12*, *17*, *35*). To assess whether the localization mechanism of the trimeric metazoan Mon1-Ccz1 containing complexes may be conserved, we investigated the experimental structure of *Dm*Mon1-Ccz1-Bulli (*14*, *16*) and AlphaFold models (*36*) of human (*Hs*) Mon1-Ccz1-RMC1 and *Xenopus laevis* (*Xl*) Mon1-Ccz1-Bulli (fig. S8, B to G). The analysis of the coulomb potential of the membrane binding interface of the different metazoan complexes reveals that the prominent basic patch of Mon1 is not conserved (Fig. 3A), suggesting that lipid interactions of these complexes may be weaker than those of *Ct*Mon1-Ccz1. Of note, clusters of basic residues are located on the β-propeller of Bulli/RMC1 (Fig. 3A), which interestingly are conserved (*14*). We thus asked to what extent direct lipid binding as a recruitment mechanism can also be observed for the metazoan Rab7 GEF homologs. A weak interaction of *Dm*Mon1-Ccz1-Bulli with a supported bilayer could be detected by QCM-D (Fig. 3, D and E). For the trimeric complex from flies, we could calculate a *K*_D_ of ~1.5 μM, which is almost 25 times weaker than for *Ct*Mon1-Ccz1. With QCM-D, we could also detect weak membrane binding of trimeric *Xl*Mon1-Ccz1-Bulli (Fig. 4A). Because the protein was not stable at concentrations >2.5 μM needed to reach saturation in a titration experiment, we could not calculate the *K*_D_ of this interaction.

### A basic patch of the Bulli β-propeller domain binds to anionic membranes and is required for its function in flies

Because the basic patch on Mon1 is less pronounced in the metazoan GEF complexes while RMC1/Bulli provides an alternative potential membrane binding site, we hypothesized that RMC1/Bulli may have an important function in localization. To test this, we first purified dimeric *Xl*Mon1-Ccz1 for QCM-D measurements. No binding of the dimer could be detected, suggesting that Bulli is needed for membrane interaction (Fig. 4B).

The basic patch of Bulli is located on the WD40 β-propeller domain and is primarily formed by four lysine residues (Fig. 4C). We thus generated an *Xl*Bulli charge inversion mutant 4xKE with these four lysines changed to glutamate (K141E, K147E, K188E, and K191E). This mutant also showed loss of membrane binding in QCM-D measurements, consistent with a role of the Bulli basic patch in membrane binding (Fig. 4D). Together, these results demonstrate a conserved membrane-binding behavior of metazoan Mon1-Ccz1-Bulli/RMC1 complexes compared to the fungal Mon1-Ccz1 complexes that, however, requires the auxiliary third subunit Bulli/RMC1 rather than Mon1 in vitro.

**Fig. 5.**
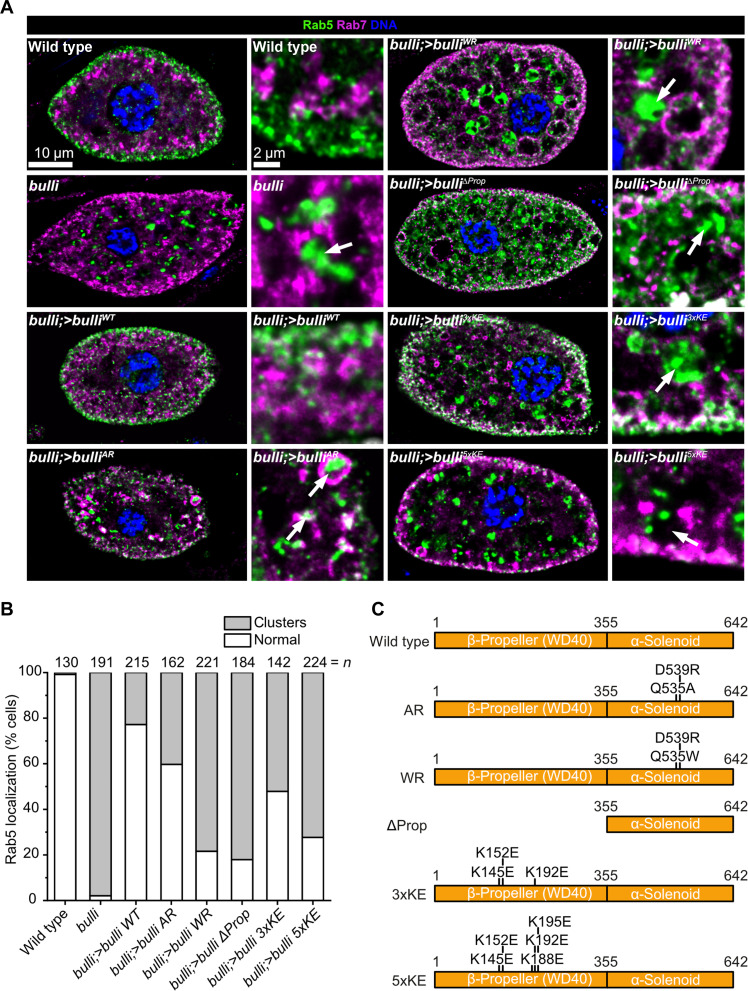
Mechanistic dissection of Bulli function for endosomal maturation in flies. (**A**) Immunohistochemistry staining of Rab5 (green) and Rab7 (magenta) localization in fly nephrocytes from WT, *bulli* knockouts, and animals reconstituted with different constructs. Nuclear staining was performed with 4′,6-diamidino-2-phenylindole (blue). (**B**) Ratio of cells with normal Rab5 distribution and cells with intracellular Rab5 clusters of different genotypes. (**C**) Schematic representation of the different *bulli* variants used in the reconstitution experiments. (AR: Q535A and D539R; WR: Q535W and D539R; ΔProp: deletion of amino acids 1 to 354; 3xKE: K145E, K152E, and K192E; 5xKE: K145E, K152E, K188E, K192E, and K195E).

To investigate the physiological relevance of the characterized interactions, we turned to *D. melanogaster* as a model system and analyzed nephrocytes from different transgenic flies. Nephrocytes are scavenger cells that clean the internal body fluid (hemolymph) of the animals and, for this purpose, have a highly active endocytic system (*37*, *38*). Wild-type nephrocytes show a high density of Rab5-positve early endosomes peripheral at the plasma membrane, and Rab7-positive late endosomes located toward the center of the cell (Fig. 5A). Deletion of the *bulli* gene results in a disruption of the endocytic pathway and defective organization of the endolysosmal system (Fig. 5A) (*8*). Notably, Rab5 localizes to clusters of membrane structures throughout the cell instead of early endosomes in the cell periphery, which we used for scoring of the phenotype (Fig. 5, A and B). Reintroduction of *bulli^WT^* rescued the phenotype as observed by reduction of Rab5 clusters and increase of normal Rab5 localization (Fig. 5B). We next tested two mutants *bulli^AR^* (Q535A and D539R) and *bulli^WR^* (Q535W and D539R) that disrupt the interface between the Bulli α-solenoid domain and Ccz1 (Fig. 5C) (*14*). The constructs show impaired functionally that correlates well with the differential reduction of binding observed previously (*14*), with the *bulli^WR^* representing the more severely affected variant. Thus, the function of Bulli is directly linked to Mon1 and Ccz1.

To address the role of the basic patch, we first investigated a deletion of the β-propeller *bulli*^Δ*Prop*^ (lacking amino acids 1 to 354) (Fig. 5C). This also led to severe mislocalization of Rab5, demonstrating that the domain conveys the function of Bulli in the context of the trimeric RabGEF (Fig. 5, A and B). More specifically, we generated two charge inversion mutants *bulli^3xKE^* (K145E, K152E, and K192E) and *bulli^5xKE^* (K145E, K152E, K188E, K192E, and K195E) in the basic patch (Fig. 5C), which were designed in analogy to the construct *Xl*Bulli 4xKE that we characterized in vitro (Fig. 4, C and D). Disruption of the basic patch had a detrimental effect on Rab5 localization, expectedly more pronounced for the 5xKE than the 3xKE variant (Fig. 5, A and B). These data demonstrate that the basic patch that binds anionic lipids in vitro is required for the function of Bulli in physiology, suggesting that Bulli is a key component for membrane targeting of metazoan Mon1-Ccz1-Bulli complexes.

### Membrane recruitment of Fuzzy-Inturned by a BAR domain protein adaptor

**Fig. 6.**
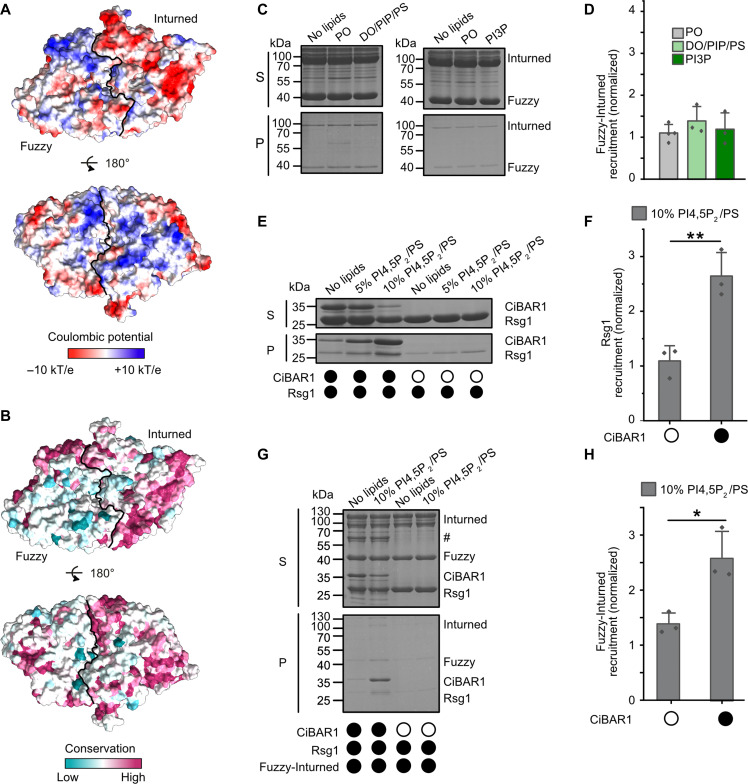
Membrane recruitment of Fuzzy-Inturned. (**A**) Surface representation of Fuzzy-Inturned colored by electrostatic potential and (**B**) conservation in two opposing views. (**C**) Sedimentation assay of Fuzzy-Inturned with no, neutral PO (81 mol% PO-PC, 18 mol % PO-PE, and 1 mol% DP-PE-Atto565), charged DO/PIP/PS (73 mol% DO-PC, 18 mol% DO-PE, 1 mol% DP-PE-Atto565, 2 mol% PI3P, 1 mol % PI(3,5)P_2_, and 5 mol% PS) liposomes, and liposomes with high PI3P (76 mol% PO-PC, 18 mol% PO-PE, 1 mol% DP-PE-Atto565, and 5 mol% PI3P). (**D**) Quantification of (C) normalized to no lipid control from *n* = 3 independent experiments. (**E**) Sedimentation assay of Rsg1 and a CiBAR1-Rsg1 complex with liposomes containing varying amounts of PI(4,5)P_2_. (**F**) Quantification of Rsg1 binding to 10% PI(4,5)P_2_/PS liposomes (61 mol% PO-PC, 18 mol% PO-PE, 1 mol% DP-PE-Atto565, 10 mol% PI(4,5)P2, and 10 mol% PS) in the absence or presence of CiBAR1 normalized to no-lipid control from *n* = 3 independent experiments. (**G**) Sedimentation assay of Fuzzy-Inturned and Rsg1 or CiBAR1-Rsg1 with 10% PI(4,5)P_2_/PS liposomes. “#” indicates protease from purification. (**H**) Quantification of (G) normalized to no-lipid control from *n* = 3 independent experiments. Sedimentation assays were analyzed by SDS-PAGE and Coomassie staining. Bar graphs represent means ± SD. Statistical analysis was performed by unpaired *t* test, *P < 0.05 and **P < 0.01.

The membrane targeting mechanism of Fuzzy and Inturned has not been studied in detail, but both proteins have been reported to interact with monophosphorylated PIPs—with a preference for PI3P—in protein-lipid overlay assays (*25*). However, analysis of the surface properties of the Fuzzy-Inturned complex reveals a mixed distribution of positive and negative charges and no conserved basic or hydrophobic patches (Fig. 6, A and B, and fig S9). We therefore tested the membrane binding of Fuzzy-Inturned in liposome sedimentation assays. Fuzzy-Inturned does bind neither liposomes generated from a neutral lipid mix that has few packing defects (PO) nor charged liposomes with increased packing defects (DO/PIP/PS) (Fig. 6, C and D). We used Mon1-Ccz1 as a control (*13*, *17*), which does not associate with neutral liposomes but is robustly recruited to charged liposomes with packing defects (fig. S10, A and B). We also could not detect any interaction of Fuzzy-Inturned with liposomes that have a high content of 5% PI3P (Fig. 6, C and D). Our findings are not consistent with the previously suggested function of Inturned and Fuzzy as PIP-binding proteins (*25*), pointing to alternative mechanisms.

Recruiter proteins play a key role in the localization of RabGEF complexes (*12*, *29*, *39*). From the notion that RabGEFs are frequently recruited by upstream GTPases in trafficking pathways the concept of Rab cascades has emerged (*40*). In the family of TLD GEFs, the GTPase Rab5 is described as recruiter for Mon1-Ccz1(-Bulli) (*14*, *41*) and Rab9 for the BLOC-3 complexes (*22*, *42*). Thus, the Fuzzy-Inturned complex may also be localized to its target compartments by interactions with recruiter proteins that enable tight membrane binding. It is therefore tempting to speculate that the GTPase Rsg1, which binds Fuzzy-Inturned (*24*), may serve as recruiter for this complex. However, Rsg1 does not contain a lipidation site or any other membrane anchoring motif. Consistent with this notion, we also did not observe a direct interaction of Rsg1 with liposomes containing packing defects and PIPs in sedimentation assays (Fig. 6, E and F).

A recent study reported an interaction of Rsg1 with the classical BAR domain protein CiBAR1 (*28*), which forms a curved helix dimer and interacts with charged lipids (fig. S10C) (*26*, *27*). Thus, CiBAR1 may be an adaptor that links Fuzzy-Inturned via Rsg1 to membranes. We coexpressed CiBAR1 and Rsg1 and were able to purify a complex of both proteins with equimolar stoichiometry as judged from a Coomassie-stained SDS–polyacrylamide gel electrophoresis (SDS-PAGE) gel (fig. S10D). The recombinant CiBAR1-Rsg1 complex was efficiently recruited to PI(4,5)P_2_ containing liposomes (Fig. 6, E and F), consistent with the PIP specificity reported for CiBAR1 before (*27*). Furthermore, Fuzzy-Inturned could be recruited to the liposomes by CiBAR1 and Rsg1 but not by Rsg1 alone (Fig. 6, G and H). Thus, CiBAR1 serves as an adaptor protein for the mammalian CPLANE complex.

**Fig. 7.**
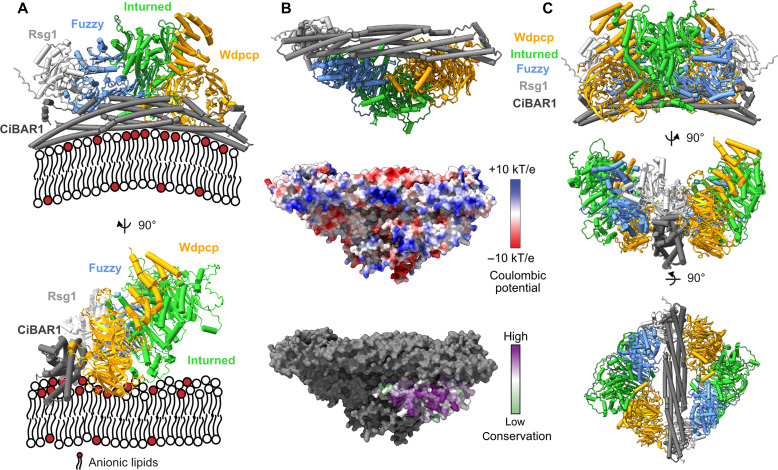
Model for CPLANE membrane recruitment. (**A**) AlphaFold3 prediction of a CPLANE (Rsg1-Fuzzy-Inturned-Wdpcp)-CiBAR1 complex in two perpendicular orientations positioned on a lipid bilayer as defined by the membrane interaction interface of CiBAR1. (**B**) Membrane-facing view of CPLANE-CiBAR1 in cartoon representation, as surface representation with electrostatic potential and as surface representation with Wdpcp colored by conservation. (**C**) Model of a decameric complex containing two CPLANE complexes docked onto a CiBAR1 dimer.

On the basis of these findings, we created a model of a CiBAR1-bound CPLANE complex using AlphaFold3 (fig. S10, E to G) (*36*). All subunits Rsg1, Fuzzy, Inturned, and Wdpcp are predicted to bind above or at the putative membrane interaction interface as defined by the concave PIP interaction surface of CiBAR1 (Fig. 7A). In this model, the β-propeller of Wdpcp would align at the membrane with a conserved basic patch (Fig. 7B). This is reminiscent of our findings regarding Bulli and suggests that both proteins, although their structural integration in the complex is entirely different (fig. S11), may fulfill a similar function in orienting the complexes on the membrane. Because CiBAR1 is a symmetric dimer that can bind two molecules of Rsg1, we docked two CPLANE complexes to the adaptor protein, which could be accommodated without major clashes (Fig. 7C).

## DISCUSSION

Our comparative study of Mon1-Ccz1 and Fuzzy-Inturned complexes reveals that the catalytic mechanism of these RabGEFs requires a Y-K motif in their substrate GTPases. The related BLOC-3 activates GTPases with a Y and an R at the equivalent position (*20*), which defines a Y-K/R consensus motif as a requirement for TLD RabGEF substrates, as previously proposed (*15*, *22*). We identified four additional Rab GTPases with this motif at the correct position in their switch 1 region (fig. S5C). Because the GEFs for some of these Rabs are not known, they might represent previously unidentified targets for TLD RabGEFs. However, in the case of Rab7L, which has such a Y-K motif, it was already shown that it is not a substrate of any of the known TLD RabGEFs (*5*).

With respect to membrane recruitment, we observe a notable difference between the fungal Mon1-Ccz1 complexes, which strongly bind to charged lipids, and the metazoan Mon1-Czz1-Bulli homologs, which only bind weakly. Also, while the electrostatic interactions of the fungal complexes are primarily mediated by the Mon1 subunit (*43*), metazoan complexes depend on Bulli/RMC1 for membrane association in vitro (Fig. 4) and functionality in animals (Fig. 5). These findings establish a molecular function of these additional subunits only found in metazoans. Loss of Bulli/RMC1 markedly affects functionality of the endolysosomal system (*7*, *8*, *10*), but the catalytic GEF function of Mon1-Ccz1 is largely retained in the absence of a third subunit (*8*) in vitro. Our mutational analysis reveals a supporting role in membrane recruitment as function of Bulli/RMC1, which explains the observed phenotypes and adds another layer of spatiotemporal control during endosomal maturation.

Our work does not confirm a PIP specificity of Fuzzy and Inturned, as reported in a previous study that used protein-lipid overlay assays (*25*), nor do we detect any other direct lipid interaction of Fuzzy-Inturned. The preferential interaction with PI3P may represent a false positive resulting from the caveats of this assay where lipids are spotted on a nitrocellulose membrane and are not incorporated into a lipid bilayer. Because PIP strips represent the less physiological setup, we would tentatively rely on the results from sedimentation assays and conclude that Fuzzy and Inturned do not specifically recognize PI3P.

In addition to direct lipid interactions, membrane targeting of Mon1-Ccz1 depends on the binding to lipid anchored recruiter proteins. Associations with Atg8 or Rab5 GTPases via an effector interaction contribute to autophagosomal or endosomal localization, respectively (*12*, *17*, *39*). For the metazoan Mon1-Ccz1-Bulli/RMC1 complexes, with reduced membrane affinity compared to the fungal homologs, the activity of the GEF and membrane targeting is dominated by recruiter proteins (*29*). It is therefore plausible that Fuzzy-Inturned will be regulated similarly. Our findings establish CiBAR1 as a recruiter protein for CPLANE, in line with previous cell biological studies (*28*). This protein has been linked to ciliogenesis and is a likely mediator for directing Fuzzy-Inturned activity and Rab23 to the basal body. CiBAR1 binds Chibby1 (*44*), which in turn interacts with the Rab8 GEF Rabin8 (*45*). Thus, it is tempting to speculate that the activation of two key GTPases for the progression of ciliogenesis, Rab8 and Rab23, may be coordinated.

Fuzzy-Inturned is linked to its membrane recruiter protein via the small GTPase Rsg1. The role of Rsg1 in ciliogenesis and as part of CPLANE is established (*24*, *46*, *47*), but little is known about its molecular function. While Rsg1 engages Fuzzy in an effector mode in its GTP-bound form (*25*), the computational model indicates that the interaction with CiBAR is nucleotide independent (fig. S10H). This contrasts with other examples of BAR domain interactions with Rho or Rab GTPases, which require the active, GTP-loaded GTPases (*48*, *49*). It will be interesting to investigate whether a GEF and/or GAP for Rsg1 exist that may regulate its nucleotide loading state. The interaction between CiBAR1 and Rsg1 should thereby not be affected, but the binding to Fuzzy and thus the membrane targeting of CPLANE could potentially be regulated.

Because Fuzzy-Inturned is not only implicated in ciliogenesis, it is possible that additional adaptors besides CiBAR1 can direct Fuzzy-Inturned to other locations. For example, the recruitment of the complex in planar cell polarity signaling may involve different mechanisms. The identity of the required factors involved in this pathway remains to be investigated.

Our proposed model of a fully assembled membrane-associated CiBAR1-CPLANE complex predicts that a conserved basic patch of the subunit Fritz may support membrane binding. This is reminiscent of our findings regarding Bulli, which is a structural homolog of Fritz. The structural integration of Fritz and Bulli within their respective complexes is entirely different (fig. S11), which could point to an adaptation to accommodate a similar function in orienting the complexes on the membrane. This observation further supports the main conclusions of our study, which reveal mechanisms of molecular fine-tuning in TLD family GEF complexes to achieve specialization for dedicated functions.

## MATERIALS AND METHODS

### Protein expression and purification

#### HsFuzzy-Inturned-Rab23^N121I^

The complex of human FLAG-Inturned, His-SUMO-Fuzzy, and nucleotide-free glutathione *S*-transferase (GST)–Rab23^N121I^ was produced in *Spodoptera frugiperda* 21 cells using the biGBac expression system (*50*). Cells were lysed in buffer A [100 mM tris (pH 8), 200 mM NaCl, 0.5 mM tris(2-carboxyethyl)phosphine (TCEP), and 1 mM MgCl_2_] supplemented with 0.25% Igepal CA-630, deoxyribonuclease I (DNase I; 0.025 mg/ml), and protease inhibitor mix HP (Serva). Complexes were isolated on glutathione agarose and eluted by proteolytic cleavage of the GST affinity tag. Proteins were further purified via size exclusion chromatography (SEC650 10/300, Bio-Rad) with buffer B [50 mM tris, 200 mM NaCl, 0.5 mM TCEP, and 1 mM MgCl_2_ (pH 8)].

#### CtMon1-Ccz1^ΔL^-Ypt7^N125I^

*E. coli* Bl21(DE3) cells were cotransformed with GST-PreScission-Mon1, His6-SUMO-Ccz1^ΔL^ (lacking residues 360 to 460), and nucleotide-free GST-tobacco etch virus (TEV) protease site-Ypt7^N125I^, and expression (16 hours, 16°C) was induced after 30-min cold shock by 0.25 mM isopropyl-β-d-thiogalactoside. Cells were lysed in buffer C [50 mM NaH_2_PO_4_, 500 mM NaCl, 1 mM MgCl_2_, and 5% (v/v) glycerol (pH 7.3)] supplemented with protease inhibitor mix HP (Serva), DNase I (0.025 mg/ml), and lysozyme (1 mg/ml). Cleared lysates were incubated with glutathione agarose, the resin was washed, and affinity tags were cleaved off Mon1 and Ccz1. After additional washing steps, the complex was eluted by TEV cleavage of the GST tag on Ypt7^N125I^. Proteins were further purified by size exclusion chromatography (SEC650 10/300, Bio-Rad) with buffer D [25 mM Hepes, 250 mM NaCl, 1 mM MgCl_2_, and 0.5 mM TCEP (pH 7.3)].

#### Rab23 variants

*E. coli* Bl21(DE3) cells were transformed with GST-Rab23 variants (WT, K39A, Y38R, and I33A/T35A) cloned into pCDF6P vectors, and expression (16 hours, 16°C) was induced after 30-min cold shock by 0.25 mM isopropyl-β-d-thiogalactoside. Cells were lysed in buffer C [500 mM NaCl, 50 mM NaH_2_PO_4_, 5% glycerol, 2 mM dithiothreitol (DTT), and 1 mM MgCl_2_ (pH 7.5)] supplemented with protease inhibitor mix HP (Serva), DNase I (0.025 mg/ml), and lysozyme (1 mg/ml). Cleared lysates were incubated with glutathione agarose, the resin was washed, and proteins were eluted by addition of 20 mM glutathione and 10 mM DTT. Proteins were further purified by size exclusion chromatography (SEC650 10/300, Bio-Rad) with buffer D [200 mM NaCl, 10 mM Hepes, 1 mM MgCl_2_, 5% glycerol, and 0.5 mM TCEP (pH 7.3)].

#### HsFuzzy-Inturned LD1 dimer

*E. coli* Bl21(DE3) cells were cotransformed with GST-Inturned^LD1^ and His6-SUMO-Fuzzy^LD1^, and expression (16 hours, 16°C) was induced after 30-min cold shock by 0.25 mM isopropyl-β-d-thiogalactoside. Cells were lysed in buffer C [500 mM NaCl, 50 mM NaH_2_PO_4_, 5% glycerol, 2 mM DTT, and 1 mM MgCl_2_ (pH 7.5)] supplemented with protease inhibitor mix HP (Serva), DNase I (0.025 mg/ml), and lysozyme (1 mg/ml). Cleared lysates were incubated with glutathione agarose, the resin was washed, and proteins were eluted by addition of 20 mM glutathione and 10 mM DTT. Proteins were further purified by size exclusion chromatography (SEC650 10/300, Bio-Rad) with buffer D [200 mM NaCl, 10 mM Hepes, 1 mM MgCl_2_, 5% glycerol, and 0.5 mM TCEP (pH 7.3)].

#### CtMon1-Ccz1

*E. coli* Bl21(DE3) cells were transformed with pCDF6P *Ct*Mon1 and pET28HS *Ct*Ccz1, and expression (16 hours, 16°C) was induced after 30-min cold shock by 0.25 mM isopropyl-β-d-thiogalactoside. Cells were lysed in buffer C [500 mM NaCl, 50 mM NaH_2_PO_4_, 5% glycerol, 2 mM DTT, and 1 mM MgCl_2_ (pH 7.5)] supplemented with protease inhibitor mix HP (Serva), DNase I (0.025 mg/ml), and lysozyme (1 mg/ml). Cleared lysates were incubated with glutathione agarose, the resin was washed, and His-Sumo tag was proteolytically cleaved off Ccz1. The resin was further washed, and the protein complex was lastly eluted by removal of the GST tag of Mon1 through incubation with PreScission protease.

Proteins were further purified by size exclusion chromatography (SEC650 10/300, Bio-Rad) with buffer E [250 mM NaCl, 25 mM Hepes, 1 mM MgCl_2_, and 0.5 mM TCEP (pH 7.3)].

#### XlMon1-Ccz1-Bulli and XlMon1-Ccz1

Complexes were produced in *S. frugiperda* 21 cells using the biGBac expression system (*50*). Cells were lysed in buffer F [50 mM Hepes-KOH (pH 7.8), 0.5 mM EDTA, 150 mM KOAc, 1 mM DTT, and 10% Glycerol] supplemented with 0.1% NP-40, DNase I (0.025 mg/ml), and protease inhibitor mix HP (Serva). Complexes were isolated on a streptactin matrix and eluted with 2.5 mM d-desthiobiotin. Affinity tags were cleaved by TEV protease incubation, and proteins were further purified via size exclusion chromatography (SEC650 10/300, Bio-Rad) with buffer G [25 mM Hepes-NaOH (pH 7.3), 250 mM NaCl, 1 mM MgCl_2_, and 0.5 mM TCEP].

#### HsRsg1

A template plasmid encoding *Hs*Rsg1 was a gift from F. Barr, University of Oxford. *E. coli* Bl21(DE3) cells were transformed with pCDF6P hsRsg1, and expression (16 hours, 16°C) was induced after 30-min cold shock by 0.25 mM isopropyl-β-d-thiogalactoside. Cells were lysed in buffer H [200 mM NaCl, 20 mM Hepes (pH 8), 1 mM DTT, and 1 mM MgCl_2_] supplemented with protease inhibitor mix HP (Serva), DNase I (0.025 mg/ml), and lysozyme (1 mg/ml). Cleared lysates were incubated with glutathione agarose, the resin was washed, and proteins were eluted by incubation with PreScission.

#### HsCiBAR1-Rsg1

Flag-CiBAR1 was a gift from K.-I. Takemaru (Addgene, plasmid #200440) (*44*). The CiBAR1 insert was subcloned into a pET28HS vector with a His6-SUMO tag. *E. coli* Rosetta (DE3) pLysS cells were cotransformed with pET28HS-CiBAR1 and pCDF6P-Rsg1 constructs, and expression (16 hours, 16°C) was induced after a 30-min cold shock by addition of 0.25 mM isopropyl-β-d-thiogalactoside. Cells were lysed in buffer I [150 mM NaCl, 20 mM Hepes, 1 mM DTT, and 1 mM MgCl_2_ (pH 7.4)] supplemented with protease inhibitor mix HP (Serva), DNase I (0.025 mg/ml), and lysozyme (1 mg/ml). Cleared lysates were incubated with glutathione agarose, the resin was washed, and the His-Sumo tag was proteolytically cleaved off CiBAR1. The resin was further washed, and the protein complex was eluted by removal from the GST tag of Rsg1 through incubation with PreScission protease.

### In vitro GEF assay

Guanine nucleotide exchange assays were performed by loading purified GTPases with 1.5 M excess MANT-GDP in the presence of 20 mM EDTA for 30 min at 30°C. After quenching the loading reaction with 25 mM MgCl_2_, the Rab23-MANT-GDP complex was separated from excess MANT-GDP in Nap5 columns (Cytiva). GTPases (2 μM final concentration) and the minimal GEF complex (varying concentrations between 0 and 2 μM) were mixed in buffer J [200 mM NaCl, 10 mM Hepes, 1 mM MgCl_2_, and 0.5 mM TCEP (pH 7.3)], and nucleotide exchange reaction was started by adding 0.1 mM GTP. The reaction was monitored in a microplate reader (M1000 Pro, Tecan) tracing the decrease of fluorescence at λ_em_ 448 nm (λ_ex_ 354 nm) in intervals of 30 or 60 s at 25°C. Data were fitted with a first-order exponential decay function *y* = *y*_0_ + *A* × *e*^−*x*/*t*^ to calculate *k*_obs_ = τ^−1^ (s^−1^) using OriginPro (OriginLab). Catalytic efficiency was determined by plotting *k*_obs_ against the concentration of the GEF.

### Cosedimentation assay

Lipids were mixed in chloroform and dried in a SpeedVac. The lipid film was dissolved in buffer L [25 mM Hepes, 250 mM NaCl, and 1 mM MgCl_2_ (pH 7.3)] supplemented with 5% sucrose to a final lipid concentration of 2 mM. Multilamellar lipid vesicles were generated by 5 cycles of freezing in liquid nitrogen and thawing at 56°C and stored at −80°C. Before use, multilamellar vesicles were extruded 21 times through a polycarbonate membrane to generate liposomes of 400 nm in diameter.

Proteins and liposomes were mixed in buffer L in final concentrations of 1 μM Fuzzy-Inturned or Mon1-Ccz1, 2 μM CiBAR1/Rsg1, and 0.5 mM lipids, respectively. Reactions were incubated for 20 min at room temperature, and liposomes were pelleted at 20,000*g* for 20 min at 4°C. The supernatant fraction was precipitated with acetone at −20°C, and supernatant and pellet fractions were analyzed by SDS-PAGE and Coomassie staining.

Membrane compositions are as follows: PO: 81 mol% 1-palmitoyl-2-oleoyl-sn-glycero-3-phosphocholine (PO-PC), 18 mol% 1-Palmitoyl-2-oleyl-sn-glycero-3-phosphoethanolamine (PO-PE), and 1 mol% 1,2-dipalmitoyl-sn-glycero-3-phosphoethanolamine labeled with ATTO-dye 565 (DP-PE-Atto565); DO/PIP/PS: 73 mol% DO-PC, 18 mol% DO-PE, 1 mol% DP-PE-Atto565, 2 mol% PI3P, 1 mol% PI(3,5)P_2_, and 5 mol% PS; PI3P: 76 mol% PO-PC, 18 mol% PO-PE, 1 mol% DP-PE-Atto565, and 5 mol% PI3P; 5% PI(4,5)P_2_/PS: 71 mol% PO-PC, 18 mol% PO-PE, 1 mol% DP-PE-Atto565, 5 mol% PI(4,5)P_2_, and 5 mol% PS; 10% PI4,5P_2_/PS: 61 mol% PO-PC, 18 mol% PO-PE, 1 mol% DP-PE-Atto565, 10 mol% PI(4,5)P_2_, and 10 mol% PS.

### Quartz crystal microbalance with dissipation

QCM-D measurements were performed essentially as previously described (*51*, *52*). Multilamellar vesicles were freshly extruded to 100 nm through a polycarbonate membrane.

QCM-D measurements were performed with a QSense Analyzer (Biolin Scientific, Gothenburg, Sweden) and SiO_2_-coated sensors (QSX 303, 50-nm SiO_2_, 4.95 MHz) equipped with four temperature-controlled flow cells. Measurements were performed at a working temperature of 23°C with a peristaltic pump (Ismatec, Grevenbroich, Germany) with a flow rate of 75 μl/min for SLB formation and 35 μl/min for protein binding. The sensors were activated by an 11-min treatment with a ultraviolet/ozone cleaner (Ossila, Sheffield, UK) before SLB formation. For SLB formation, freshly prepared SUVs were diluted to 0.1 mg/ml with citrate buffer [10 mM tri-sodium citrate, 150 mM NaCl, and 10 mM CaCl_2_ (pH 4.6)] and flushed into the chamber. For protein binding, reaction buffer [25 mM Hepes-NaOH, 250 mM NaCl, 1 mM MgCl_2_, and 0.5 mM TCEP (pH 7.3)] was used. Proteins were titrated in increasing concentrations until a stable baseline was reached. The change in frequency (ΔΔ*F*) of the fifth overtone resonance frequency channel was plotted against the protein concentration. Nonlinear curve fitting assuming one site-specific binding was performed in OriginPro (OriginLab) to determine the *K*_D_.

### Cryo-EM sample preparation

Purified *Ct*Mon1-Ccz1^ΔL^-Ypt7^N125I^ (~0.8 mg/ml) and *Hs*Fuzzy-Inturned-Rab23^N121I^ (~0.7 mg/ml) complexes were applied to glow-discharged CF-1.2/1.3-3 Cu-50 grids. A total of 0.002% lauryl maltose neopentyl glycol was added to *Hs*Fuzzy-Inturned-Rab23^N121I^ on grid before freezing to overcome preferred orientation of particles at the air water interface. Samples were plunge frozen in liquid ethane using a Vitrobot Mark IV (Thermo Fisher Scientific). Data were collected on a Glacios electron microscope equipped with a Selectris energy filter and a Falcon 4i direct electron detector (Thermo Fisher Scientific) at 165,000-fold nominal magnification.

### Cryo-EM image processing

All cryo-EM data were preprocessed in cryoSPARC Live, and further processing was performed in cryoSPARC v3 and v4 (figs. S1 and S3) (*53*–*55*).

#### CtMon1-Ccz1^ΔL^-Ypt7^N125I^

After micrograph curation, 25,142 micrographs with a contrast transfer function (CTF) fit better than 5 Å were included for further data analysis (fig. S1). Initial particles were selected by blob picker implemented in CryoSPARC Live, and additional particle picking was performed using cryoSPARC’s Topaz wrapper (*56*). After particle duplicate removal and particle extraction in a box size of 400 pixels (Fourier cropped to 200 pixels, resulting in a pixel size of 1.36 Å per pixel), iterative rounds of two-dimensional (2D) classification and selection were performed for all particle stacks obtained from each of the picking jobs to eliminate bad picks (fig. S1). After 2D classifications, ab initio reconstruction with six classes followed by heterogeneous refinement was performed. The heterogeneous refinement produced two classes, revealing trimeric (Mon1-Ccz1-Ypt7) or dimeric (Mon-Ccz1) particle projections. These classes were combined in nonuniform (NU) refinements and submitted to subsequent iterative 3D classification with forced hard classification enabled, which resulted in two good classes containing either the dimeric or the trimeric complexes. These two classes (525,000 and 1.27 million particles) were further refined using NU refinement in a box size of 440 pixels (without Fourier cropping). The obtained reconstructions of both populations yield a global resolution of 3.0 Å [gold-standard Fourier shell correlation (GSFSC) = 0.143].

#### HsFuzzy-Inturend-Rab23^N121I^

The data of *Hs*Fuzzy-Inturned-Rab23^N121I^ were processed accordingly (fig. S3). After micrograph curation, 5960 micrographs with a CTF fit better than 5 Å were included for further data analysis. After initial blob picking, a Topaz wrapper was trained, and a total of 220,000 particles were picked. Particle extraction was performed with a box size of 384 pixels (Fourier cropped to 96 pixels, resulting in a pixel size of 2.72 Å per pixel). Iterative rounds of 2D classification and selection were performed (fig. S3). Selected classes were used for ab initio reconstruction in three classes followed by heterogeneous and NU refinements resulting in a one class of 113,000 particles. 3D classification with forced hard classification enabled resulted in identification of a dimeric (Fuzzy-Inturned, 38,000 particles) and a trimeric (Fuzzy-Inturned-Rab23^N121I^, 60,000 particles) population, which were further refined in NU refinements at a box size of 384 pixels leading to final reconstructions of 3.7- and 3.4-Å resolution (GSFSC = 0.143), respectively.

All maps were subjected to unsupervised B-factor sharpening within cryoSPARC. No symmetry was applied during processing. All GSFSC curves, angular distribution plots, and local resolution maps were generated with cryoSPARC (figs. S1 to S4). The local resolutions of refined maps (fig. S1 and S3) were estimated in cryoSPARC and analyzed in UCSF ChimeraX (*57*).

### Model building, refinement, and validation

The structures of *ct*Mon1-Ccz1 [Protein Data Bank (PDB): 7QLA] (*13*) and Ypt7 (PDB: 5LDD) (*15*), the human CPLANE complex (PDB: 7Q3D) (*25*), and the AlphaFold prediction of Rab23 (*58*) were manually fitted as rigid bodies into maps using UCSF ChimeraX and used as a starting model. Subsequently, iterative rounds of real space refinement in PHENIX (*59*) and manual adjustments in WinCoot 0.9.8.92 (*60*) were performed. Model validation was done using MolProbity (*61*) in PHENIX. Models and maps were visualized, and figures were prepared in UCSF ChimeraX. Model refinement and validation statistics are provided in table S1.

### In vivo experiments

#### Fly husbandry and genetics

If not stated otherwise, flies were reared and cultured on standard fly medium at room temperature. Fly stocks used were OregonR-P2 (BL2376) as WT control and *bulli^1-22^*/TM6B,*Tb^1^,Hu^1^* (*8*). Expression of UAS-*bulli* constructs was driven by the heart and nephrocyte-specific driver line *hand*C-Gal4 (*62*). Rescue experiments were performed at 25°C.

#### DNA cloning and generation of UAS lines

The full-length or truncated coding sequences of *bulli* were amplified via PCR from the cDNA clone MIP05619 and subsequently cloned into the pUAST.attB shuttle vector (*63*), using standard restriction cloning. Point mutations in the *bulli* coding sequence were introduced by site-directed mutagenesis, using adequate oligonucleotides and the Q5 Site-directed mutagenesis kit (New England Biolabs). All sequences were validated by conventional Sanger sequencing. Transgenic flies were generated by BestGene Inc., Chino Hills, USA.

#### Immunostaining of larval nephrocytes

Staining and imaging were performed following standard procedures (*8*). In short, third instar larvae of the appropriate genotype were dissected from the ventral body side, and viscera was removed and fixed in 4% formaldehyde at room temperature for 1 hour. Larval filets were subsequently washed four times in BBT buffer [phosphate-buffered saline (PBS; 137 mM NaCl, 2.7 mM KCl, 8 mM Na2HPO4, and 2 mM KH2PO4, pH 7.4), 0.1% Tween 20, and 0.1% bovine serum albumin (BSA)], permeabilized in PBS + 1% Triton X-100 for 1 hour, washed again in BBT, and blocked for 1 hour in saturation buffer (PBS, 0.1% Tween 20, and 1% BSA) at room temperature. Primary antibodies were incubated overnight at 4°C. After another four washing steps, secondary antibodies were incubated for 2 hours at room temperature. Primary antibodies used were rabbit anti-Rab5 (Abcam, ab31261, RRID:AB_882240; 1:250) and mouse anti-Rab7 [Developmental Studies Hybridoma Bank (DSHB), RRID:AB_2722471; 1:20]. Fluorophore-conjugated secondary antibodies were goat anti-rabbit Cy2 (1:200) and goat anti-rabbit Cy3 (1:200). Images were acquired using a Zeiss LSM800 confocal microscope equipped with a 40× oil immersion lens (numerical aperture = 1.4). Images were taken in a central focal plane of single nephrocytes with the nucleus of the cells visible. For quantification of the phenotype, each cell was manually scored for normal Rab5 localization or defective intravesicular Rab5 accumulation.
