# Supplementary material for: Mechanistic adaptation of the metazoan RabGEFs Mon1-Ccz1 and Fuzzy-Inturned
Source: Sci Adv. 2025 Aug 27;11(35):eadx2893. doi: 10.1126/sciadv.adx2893 (PMC12383261; doi:10.1126/sciadv.adx2893)
Supplement: Supplementary file 1 — Figs. S1 to S11 Table S1 [file sciadv.adx2893_sm.pdf]

Science Advances
AAAS

Supplementary Materials for

Mechanistic adaptation of the metazoan RabGEFs Mon1-Ccz1 and Fuzzy-Inturned

Stephan Wilmes *et al.*

Corresponding author: Daniel Kümmel, daniel.kuemmel@uni-muenster.de

Sci. Adv. **11**, eadx2893 (2025)
DOI: 10.1126/sciadv.adx2893

This PDF file includes:

Figs. S1 to S11
Table S1

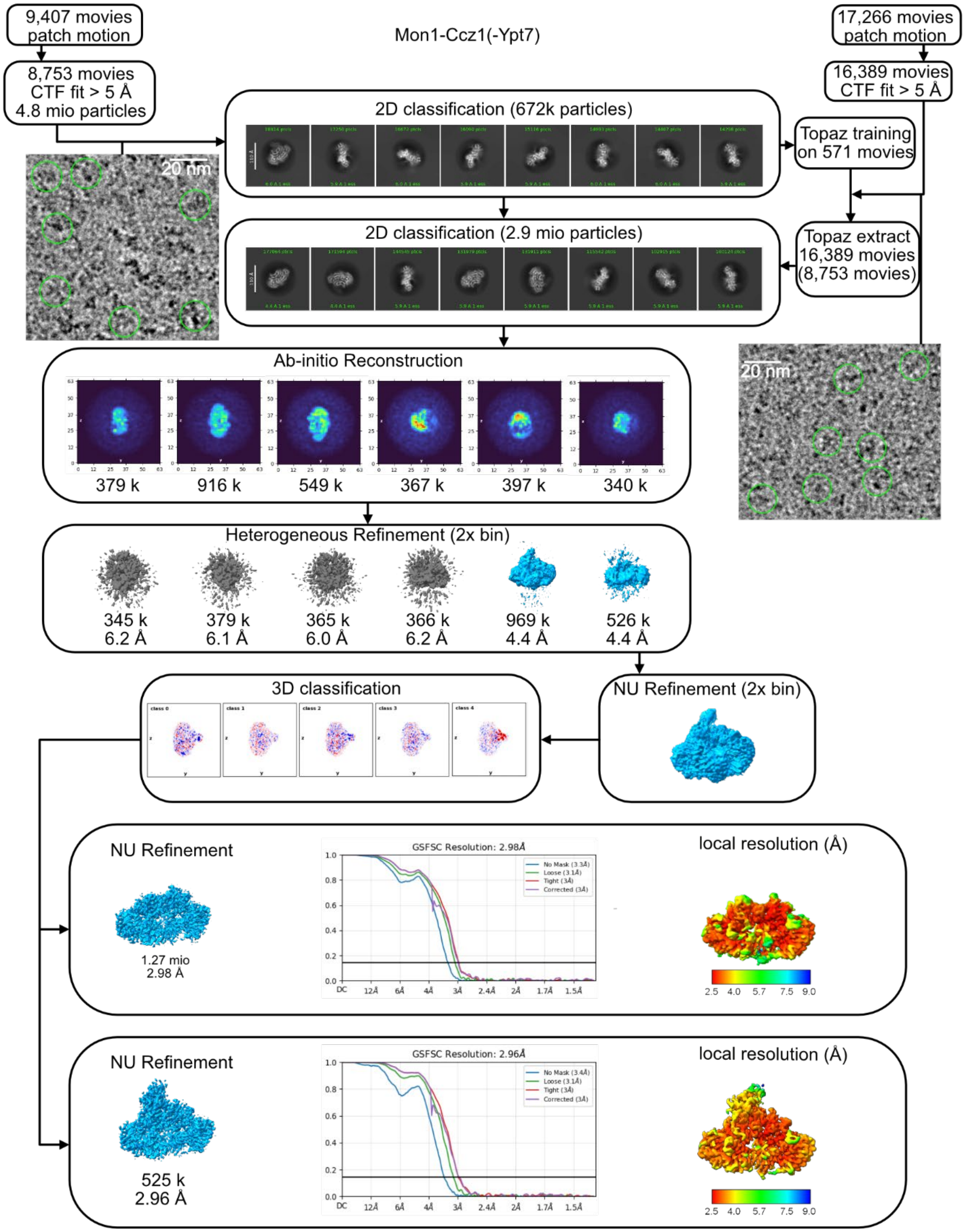

Fig. S1. Cryo-EM data processing workflow for *Ct*Mon1-Ccz1-Ypt7 and *Ct*Mon1-Ccz1.

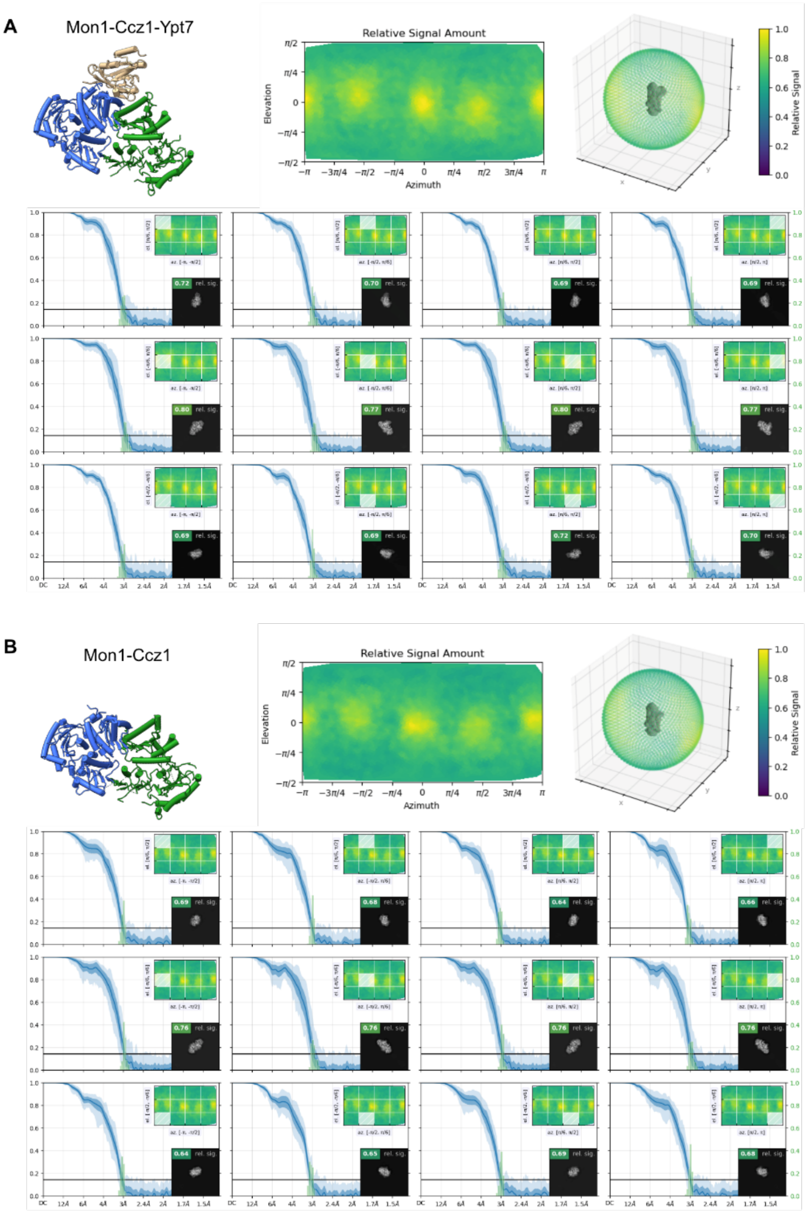

Fig. S2. Orientation diagnostics for *Ct*Mon1-Ccz1-Ypt7 (A) and *Ct*Mon1-Ccz1 (B) cryo-EM data.

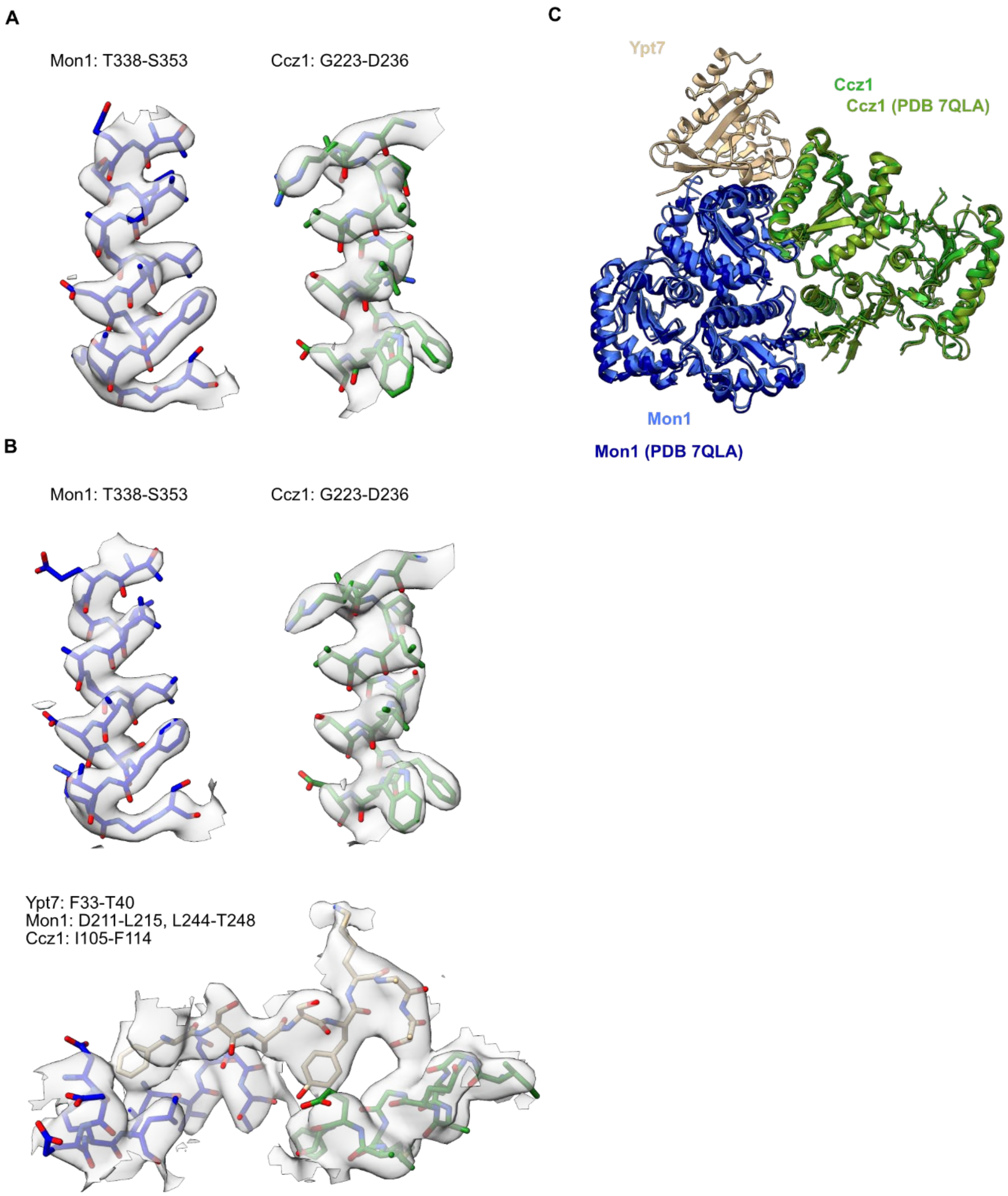

Fig. S3. Quality of the *Ct*Mon1-Ccz1 and *Ct*Mon1-Ccz1-Ypt7 structures. Model/map fit of selected areas of the (**A**) *Ct*Mon1-Ccz1 and (**B**) *Ct*Mon1-Ccz1-Ypt7 complexes. (**C**) Superposition of *Ct*Mon1-Ccz1$^{\Delta L}$-Ypt7 and *Ct*Mon1$^{\Delta N}$-Ccz1$^{\Delta L}$ (PDB ID 7QLA).

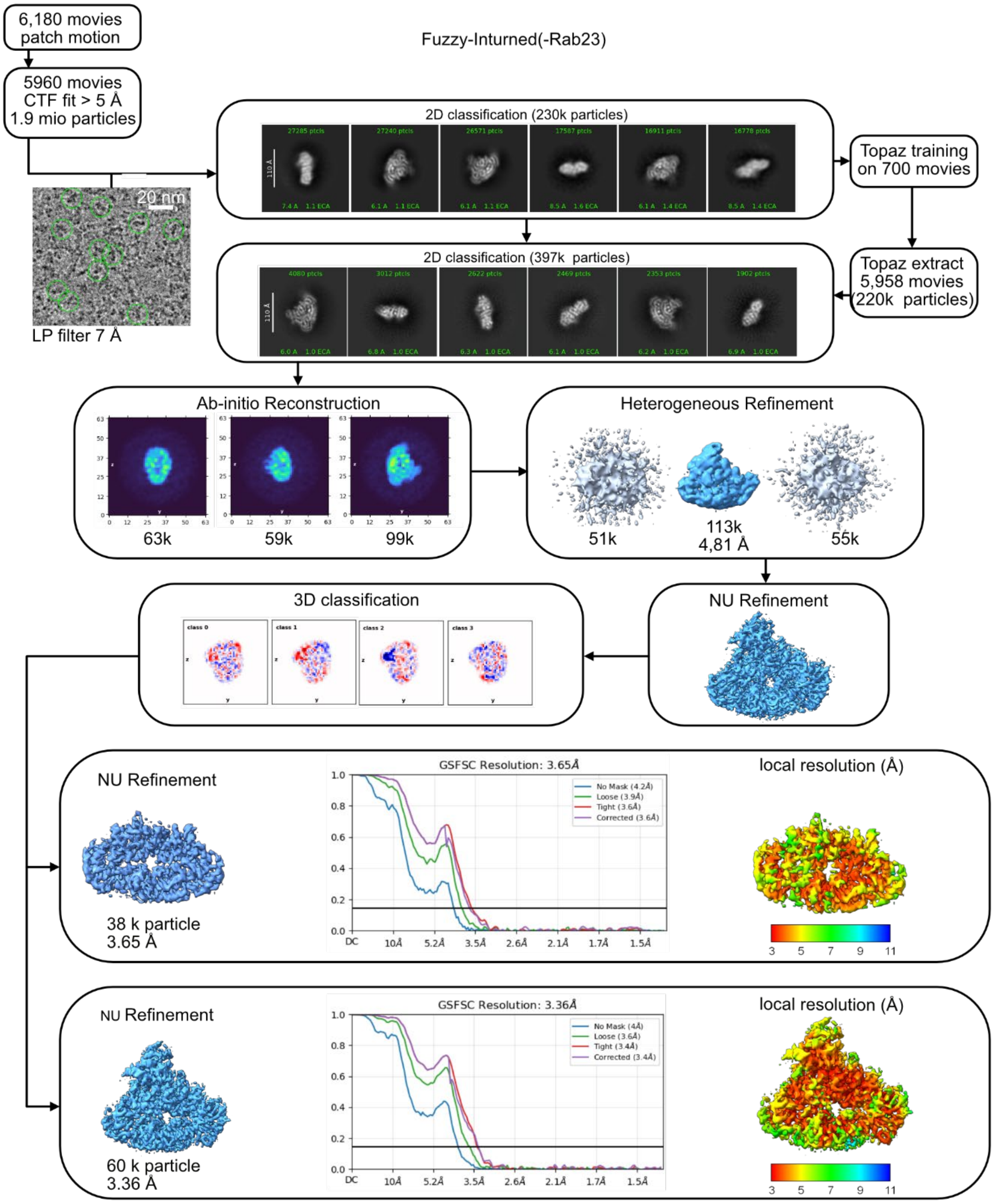

Fig. S4. Cryo-EM data processing workflow for *Hs*Fuzzy-Inturned and *Hs*Fuzzy-Inturned-Rab23.

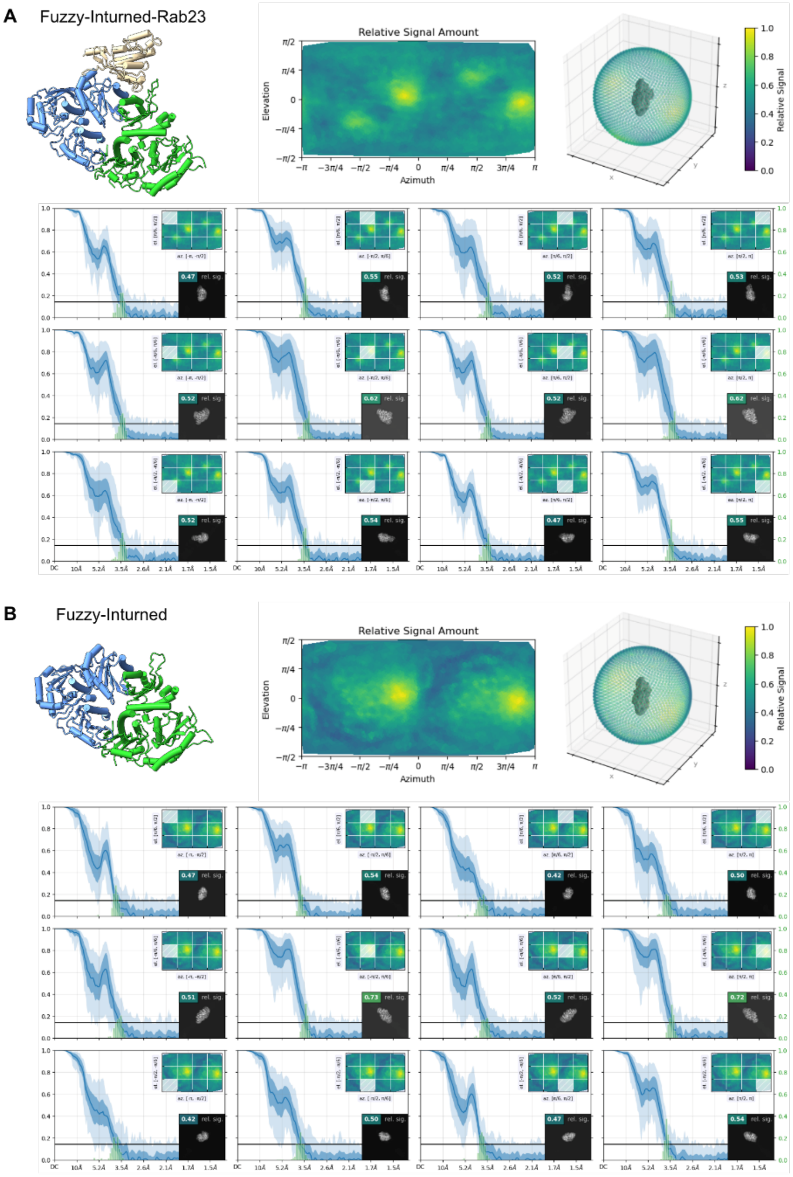

Fig. S5. Orientation diagnostics for (A) *Hs*Fuzzy-Inturned-Rab23 and (B) *Hs*Fuzzy-Inturned cryo-EM data.

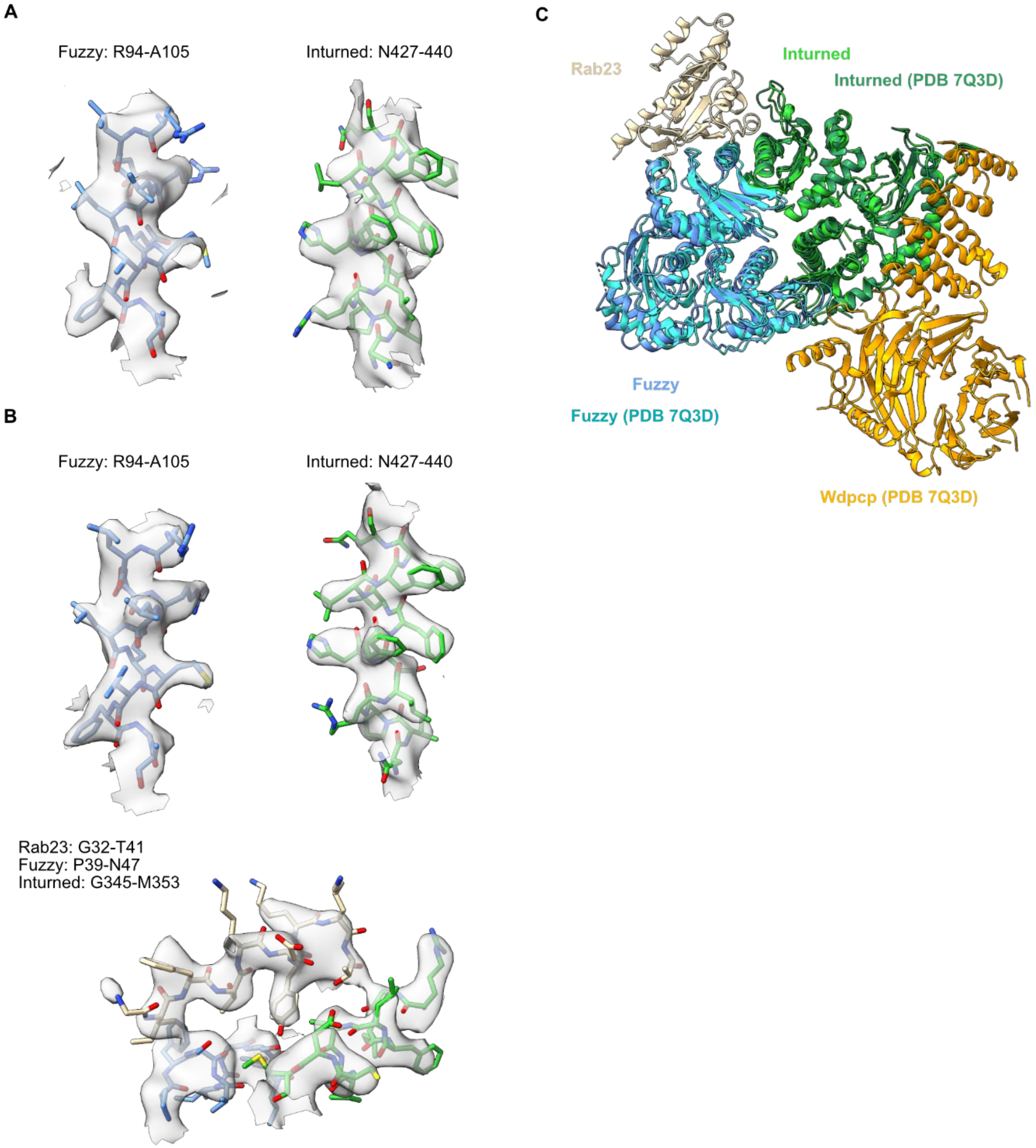

Fig. S6. Quality of the *Hs*Fuzzy-Inturned and *Hs*Fuzzy-Inturned-Rab23 structures. Model/map fit of selected areas of the (**A**) *Hs*Fuzzy-Inturned and (**B**) *Hs*Fuzzy-Inturned-Rab23 complexes. (**C**) Superposition of *Hs*Fuzzy-Inturned-Rab23 and the human CPLANE complex (PDB ID 7Q3D).

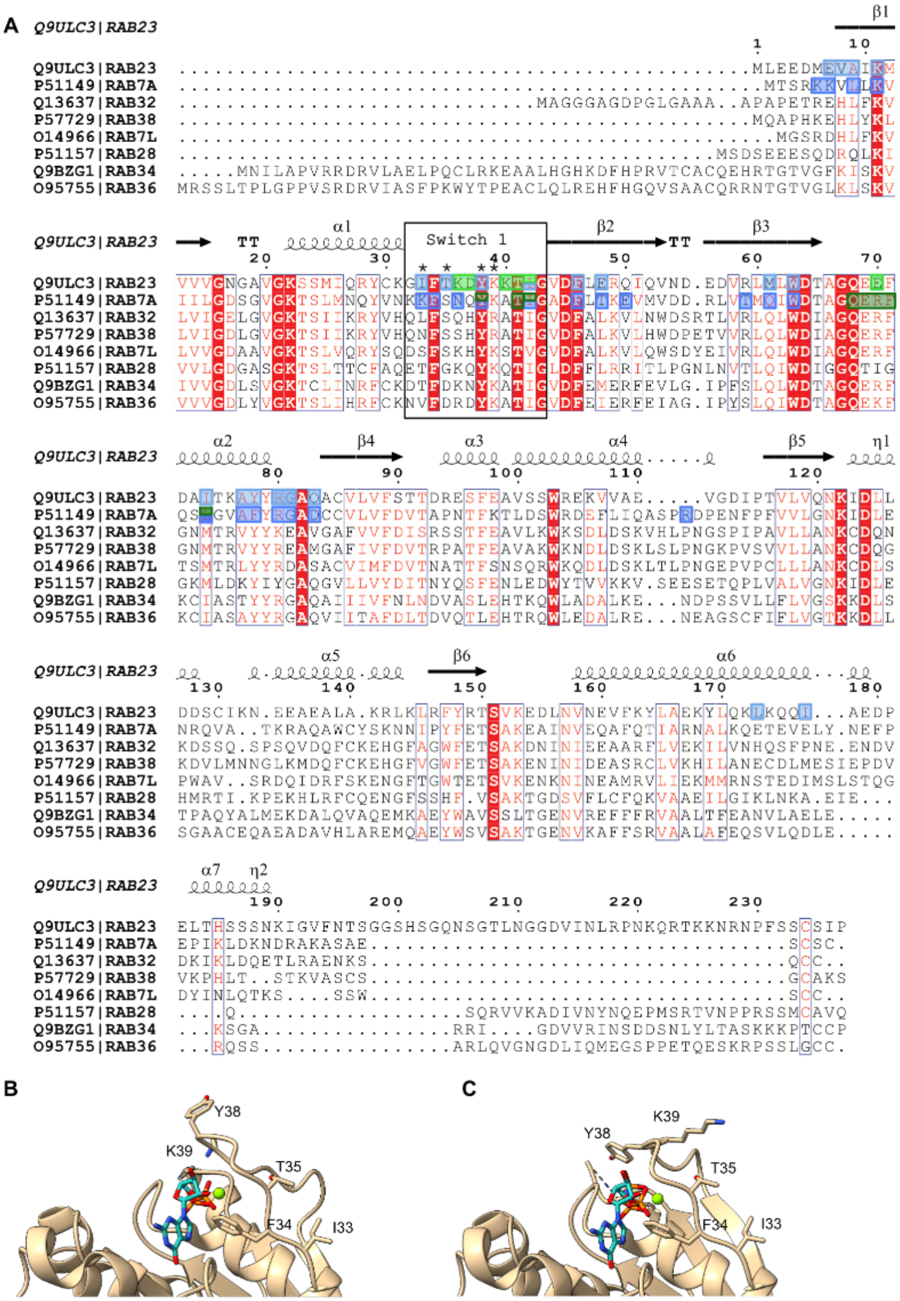

Fig. S7. Comparison of TLD RabGEF substrate GTPases. (**A**) Amino acid sequence alignment of human Rab GTPases containing FxxxYK/R motifs in their switch I. Secondary structure elements and sequence numbering are inferred from *Hs*Rab23. Residues mutated for GEF activity assays are highlighted by asterisks. Residues contacting GEF complexes are marked in blue (Mon1/Fuzzy) and green (Ccz1/Inturned). Note: Rab7 N35 differs from Ypt7 that was used for structural investigation and carries an Ala at that position. Crystal structures of hsRab23 in complex with (**B**) GDP (PDB ID 8YL3) and (**C**) the GTP analog GMPPNP (PDB ID 8YIM).

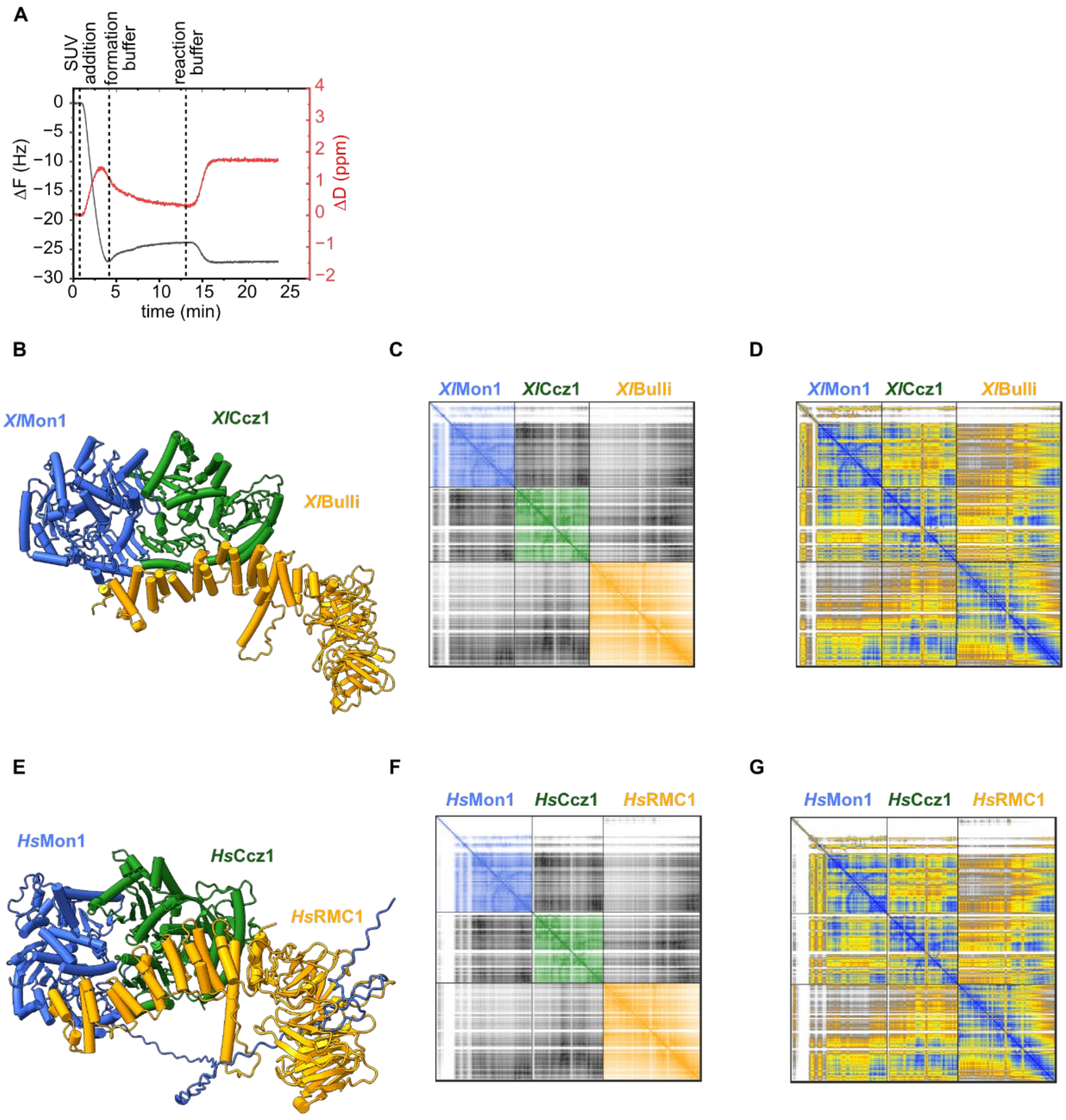

Fig. S8. QCM-D bilayer formation and modelling of metazoan Rab7GEFs. (**A**) Exemplary plot for frequency (black, deviation from resonance frequency, Δ/Hz) and dissipation (red, dissipation changes (ΔD)) shifts during bilayer formation for QCM-D measurements. Membrane composition: 74 mol% DOPC, 18 mol% DOPE, 2 mol% PI3P (18:1), 1 mol% PI3,5P2 (18:1) and 5 mol% PS. (**B**) Alphafold3 prediction of xlMon1-Ccz1-RMC1 with pTM = 0.81 and ipTM = 0.80 scores. (**C**) PAE plot for B colored by chain. (**D**) PAE plot for B colored by pLDDT. (**E**) Alphafold3 prediction of hsMon1-Ccz1-RMC1 with pTM = 0.78 and ipTM = 0.76 scores. (**F**) PAE plot for E colored by chain. (**G**) PAE plot for E colored by pLDDT.

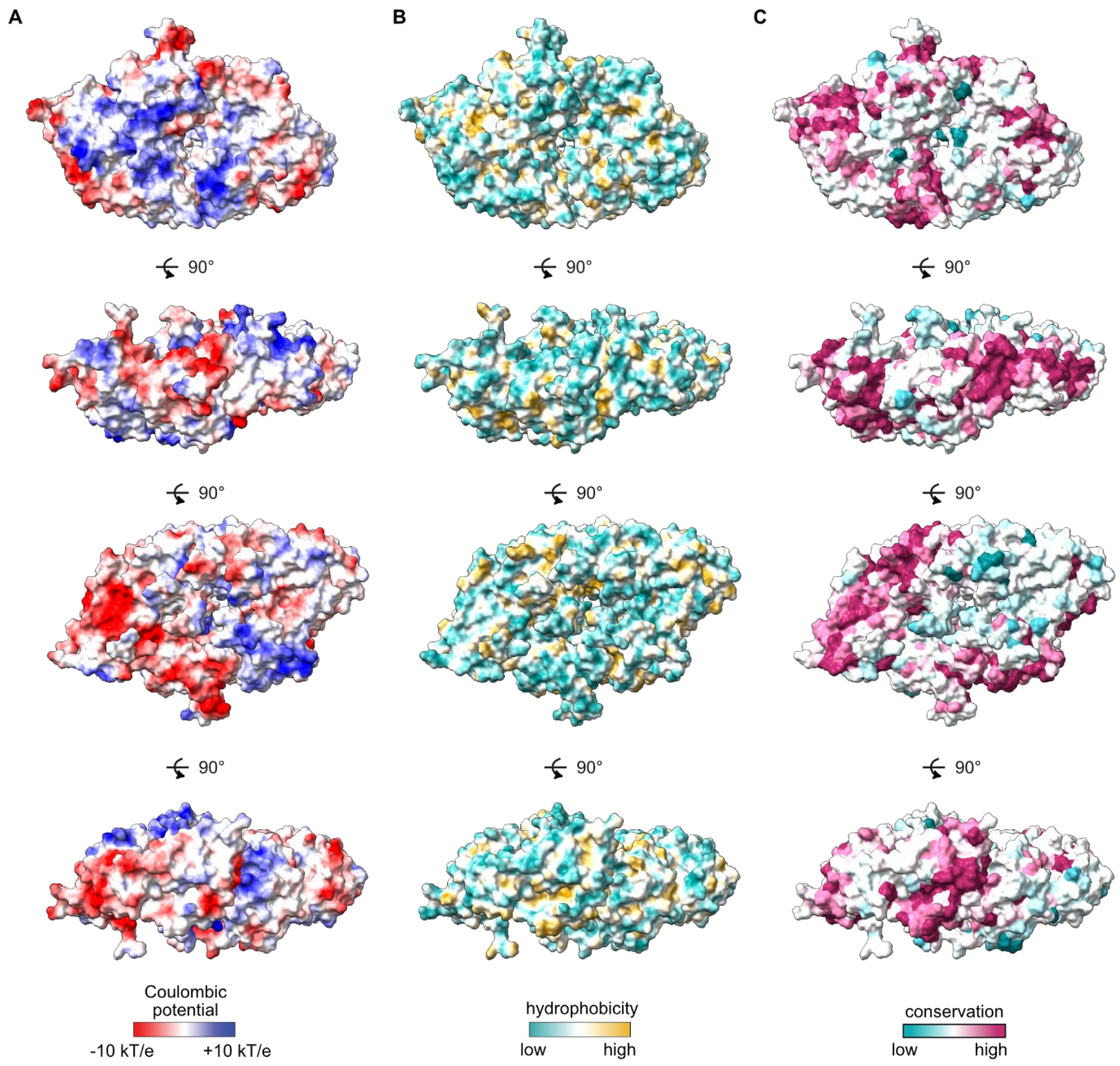

Fig. S9. Surface properties of *Hs*Fuzzy-Inturned. Surface representations of *Hs*Fuzzy-Inturned colored by (**A**) coulombic potential, (**B**) hydrophobicity and (**C**) conservation.

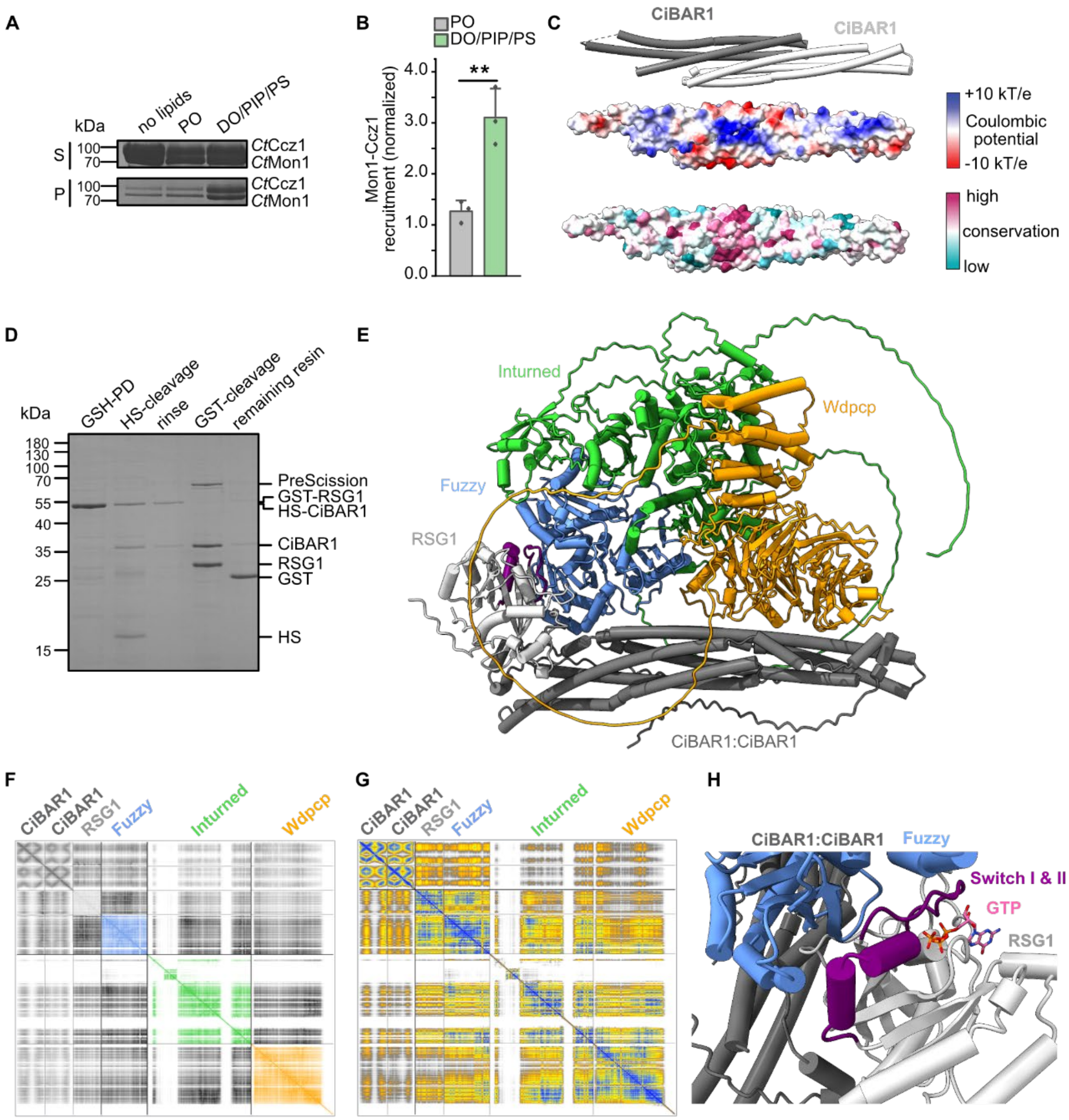

Fig. S10. CiBAR1 recruits Fuzzy-Inturned onto membranes. (**A**) The *Ct*Mon1-Ccz1 complex is recruited to membranes containing packing defects and charges in co-sedimentation assays. Lipids mixtures: PO: 81% POPC, 18% POPE, 1% DPPE-Atto 565 and DO-PIP-PS:73% DOPC, 18% DOPE, 1% DPPE-Atto 565, 2% PI3P, 1% $PI3,5P_2$, 5% PS. (**B**) Quantification of (A) from n=3 independent repeats. Bar graphs represent means ± standard deviation. Statistical analysis was performed by unpaired t-test, ** $p<0.01$. (**C**) Crystal structure of the homodimeric BAR domain protein CiBAR1 (PDB ID: 8CEG) with surface representations of the coulombic potential and conservation. (**D**) Co-purification of His-SUMO (HS) tagged CiBAR1 and glutathione-*S*-transferase (GST) tagged Rsg1. Both proteins were co-purified on glutathione resin (GSH-PD). The HS tag cleaved off CiBAR1 by incubation with SUMO protease (HS-cleavage) and protease and tag rinsed away (rinse). The complex was eluted from the resin by cleaving the GST-tag with

PreScission protease (GST-cleavage). (**E**) Alphafold3 prediction of CiBAR1(dimer) in complex with CPLANE (Rsg1, Fuzzy, Inturned, and Wdpcp) with pTM=0.71 and ipTM=0.71 scores. Rsg1 switch regions are highlighted in purple. (**F**) PAE plot of D colored by chain. (**G**) PAE plot of D colored by pLDDT scores. (**H**) Close-up of Rsg1 in the bound to CiBAR1 and Fuzzy. The bound GTP nucleotide is shown in stick representation and the switch regions of Rsg1 are highlighted in purple.

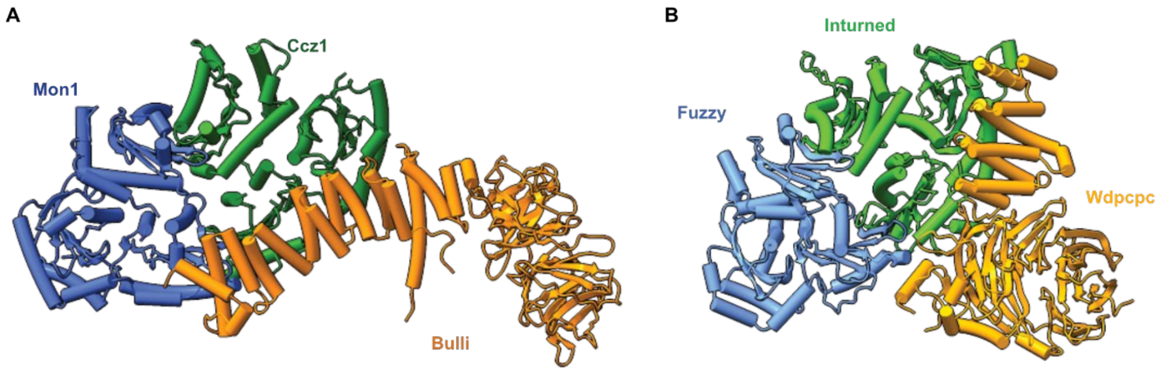

Fig. S11. Structural comparison of Mon1-Ccz1-Bulli and CPLANE complexes. (**A**) *Dm*Mon1-Ccz1-Bulli (PDB ID: 8C7G) and (**B**) *Hs*Fuzzy-Inturned-Wdpcp (PDB ID 7Q3D). Despite high structural similarities between Bulli and Wdpcp, these subunits are integrated into the complex entirely different.

Table S1. Cryo-EM data collection and refinement statistics

**Data Collection**	**Mon1-Ccz1-Ypt7**	**Mon1-Ccz1**	**IntuFy-Rab23**	**IntuFy**
Microscope	FEI Glacios		FEI Glacios	
Voltage (kV)	200		200	
Camera	Falcon 4i		Falcon 4i	
Energy filter	Selectris		Selectris	
Pixel size	0.680		0.680	
Micrographs	26,673 (25,142 final)		6180 (5690 final)	
Particles	524738,	1,271,173	60,395	37,728
Total electron does (e^{-}/ $Å^2$)	50		50	
Defocus range (µm)	-0.8 to -2.5		-0.8 to -1.8	
**Atomic model composition**				
Symmetry imposed	C1	C1	C1	C1
Non-hydrogen (protein) atoms	8253	6970	7415	6065
Residues	1052	891	938	769
**Refinement (Phenix)**				
RMSD bond length (Å)	0.003	0.003	0.003	0.003
RMSD angle (°)	0.588	0.682	0.792	0.764
Model to map fit, CC mask	0.85	0.82	0.79	0.79
Model to map fit, CC box	0.85	0.81	0.82	0.83
Resolution (FSC @ 0.143, Å)	3.0	3.0	3.4	3.7
B-factor (min/ max/ mean, $Å^2$)	46/141/71	86/242/141	75/233/129	102/236/147
**Validation**				
Clash score	14.25	9.93	18.15	17.68
Ramachandran outliers (%)	0.00	0.00	0.00	0.00
Ramachandran allowed (%)	4.02	3.70	4.96	4.83
Ramachandran favoured (%)	95.98	96.30	95.04	95.17
Molprobity score	1.94	1.77	2.50	2.45
**PDB accession**	9RS7	9RS6	9RS9	9RS8
**EMDB accession**	54212	54211	54214	54213
